# Capillary bundling of microtubules by condensates

**DOI:** 10.64898/2026.06.19.733462

**Published:** 2026-06-20

**Authors:** Bernardo Gouveia, J. Pedro de Souza, Venecia Valdez, Joshua W. Shaevitz, Howard A. Stone, Sabine Petry

**Affiliations:** 1 Department of Chemical and Biological Engineering, Princeton University, Princeton, New Jersey 08544, USA; 2 Omenn–Darling Bioengineering Institute, Princeton University, Princeton, New Jersey 08544, USA; 3 Department of Molecular Biology, Princeton University, Princeton, New Jersey 08544, USA; 4 Department of Physics, Princeton University, Princeton, New Jersey 08544, USA; 5 Lewis–Sigler Institute for Integrative Genomics, Princeton University, Princeton, New Jersey 08544, USA; 6 Department of Mechanical and Aerospace Engineering, Princeton University, Princeton, New Jersey 08544, USA

## Abstract

The cytoskeleton organizes the cellular interior using cytoskeletal filaments that rely on bundling, usually executed by stable and ordered crosslinking proteins. Bundling often requires protein complexes with at least two defined microtubule binding regions, as present in many molecular motors. Here, we establish a mechanism of microtubule bundling based on capillary forces, analogous to how wet hair sticks together. We show using *in vitro* experiments and theory that condensates can bundle microtubules through capillary forces, wherein liquid-like capillary bridges form between microtubules and adhere them together through interfacial and wetting forces. We quantify the structure and dynamics of these capillary bundles using total internal reflection fluorescence microscopy, and directly measure the charge-dependent interfacial tensions of condensates on microtubules using atomic force microscopy. Lastly, we show that these capillary bridges provide viscous resistance to motor-driven microtubule sliding that is insensitive to the bulk protein concentration. Taken together, we provide a novel mechanism for how cytoskeletal filaments bundle: through condensate-mediated capillary forces.

## I. INTRODUCTION

As part of the eukaryotic cytoskeleton, microtubules are rigid, tubular polymers that form the structural foundation of the mitotic spindle [1], the neuronal axon [2], and cilia [3]. Microtubules often form bundles with other microtubules to generate larger, more stable structures, such as kinetochore fibers [4, 5]. The standard view of how microtubule bundles form is through the action of crosslinking proteins that bridge two microtubules together through at least two definable microtubule binding regions that typically have positive charge [6–8]. Passive crosslinkers range from the physiological dimer PRC1 [9, 10] to small, synthetic peptides with multivalent, positively charged residue strings [11]. Active crosslinkers include the tetrameric motor protein kinesin-5 (Eg5/KIF11) [12, 13] and the dimeric motor protein kinesin-14 (Ncd/HSET) [13, 14]. In these examples, the mechanism of microtubule bundling is readily apparent: these proteins form stable multimers or have multivalent motifs that can directly bind two microtubules simultaneously using multiple microtubule binding regions.

There has been recent interest in studying microtubule associated proteins (MAPs) that form biomolecular condensates above a saturation concentration—that is, MAPs that can thermodynamically partition into a dilute phase and dense phase (the condensate). Even though the MAP content of the condensate is elevated, it often exhibits liquid-like properties [15, 16]. Although the focus in the literature has been on how condensed MAPs regulate active microtubule processes such as dynamic instability and microtubule nucleation, it is striking that in nearly all of these studies microtubule bundling is also observed. Examples include TPX2 [17–20], BuGZ [21], Tau [22–24], LEM2 [25], CLIP-170 [26–28], MAP65 [29, 30], TPPP [31], and CPC components [32] both *in vitro* and *in vivo*. This is not only true for microtubules; actin-binding proteins that form condensates have also been shown to bundle actin filaments, with examples including VASP [33], the Nephrin–Nck–NWASP system [34, 35], and the LAT signaling system [36]. Thus, there seems to be a general relationship between the ability of cytoskeletal-associated proteins to form condensates and their ability to bundle filaments. While this relationship has been noticed in the literature [37], the precise mechanism by which this occurs remains to be determined.

Most of these MAPs are composed of large intrinsically disordered regions with only one microtubule binding domain, if at all. They form condensates through multivalent, weak interactions [16]. Due to the seemingly generic nature of these bundling observations, it is unlikely that condensed MAPs bundle microtubules by stably dimerizing and bridging two filaments together through multiple defined binding regions. We hypothesized that a more mesoscale mechanism must be at play operating above the length scale of single protein interfaces, especially in the vicinity of or above the saturation concentration for biomolecular condensation.

Specifically, we hypothesized that condensed MAPs can bundle microtubules through capillary forces, which occur when a liquid phase wets a solid substrate and result from the interfacial tension of the liquid-liquid interface [38]. These forces can create liquid capillary bridges between two substrates, and are precisely the everyday forces we experience when a wetted finger turns the page of a book, or when wet hair sticks together, and are common in millimeter-to-micrometer scale wetted fiber networks [39, 40]. Due to the liquid-like nature of biomolecular condensates and the variety of soft substrates available to wet, such as chromatin [41, 42], cytoskeletal filaments [43–45], and membranes [46, 47], interfacial forces are already being implicated in diverse intracellular contexts that involve condensates [48].

To test this bundling hypothesis, we employed total internal reflection fluorescence microscopy (TIRFM) and atomic force microscopy (AFM) experiments on model proteins TPX2 and BuGZ *in vitro* in combination with theoretical modeling. We show that condensed TPX2 and BuGZ can bundle microtubules via liquid-like capillary bridges, and that this mechanism is fundamentally distinct from bundling via single-molecule crosslinkers.

## II. RESULTS

### A. Condensed MAPs bundle microtubules through capillary bridges *in vitro*

To study how condensed MAPs bundle microtubules, we used purified recombinant BuGZ-BFP and GFP-TPX2 as our model proteins throughout this paper. Both have been reported previously to phase separate into condensates above a bulk saturation concentration [20, 21]. However, whereas BuGZ only has one microtubule binding region [21], TPX2 has two [49], and so in principle it could bundle microtubules through direct crosslinking as well as through condensation. We performed a bulk phase separation assay and found that BuGZ condensed at ≈ 140 nM while TPX2 condensed at ≈ 50 nM in assay buffer (Fig. S1, Supplementary Methods). We note that condensation on microtubules will occur at a concentration lower than the bulk saturation concentration due to prewetting effects [45, 50], so the bulk saturation value can only be taken as a rough guide.

**FIG. 1.**
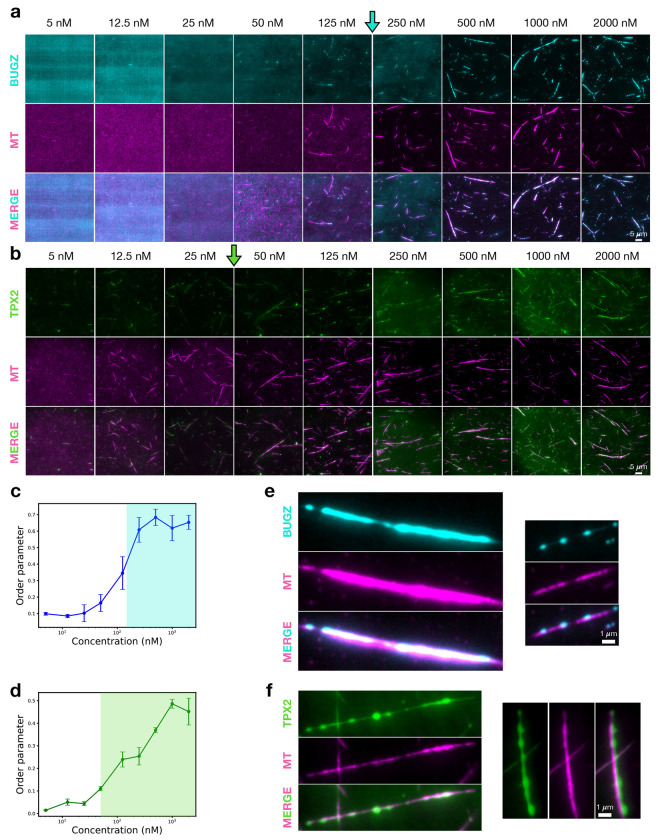
Condensed microtubule-associated proteins (MAPs) bundle microtubules. TIRFM images of microtubule bundles that sedimented to the flow channel surface after a 10 min incubation with **a.** BuGZ (1 microtubule binding region) and **b.** TPX2 (2 microtubule binding regions). Colored arrows indicate concentration above which these MAPs form condensates in bulk solution. Microtubule channel lookup tables are the same across concentrations to enable direct comparison. Note the decrease in background soluble tubulin above the phase boundaries, indicating partitioning into condensates. MAP channel look-up tables are optimized per concentration to allow for visualization. Scale bars are 5 *μ*m. Order parameter that describes the bundling transition for **c.** BuGZ and **d.** TPX2 as a function of bulk MAP concentration. The order parameter is calculated as $\frac{\int_{\Omega }\;\;S(k)dk}{\int_{-\infty }^{\infty }\;\;S(k)dk}$, where *S*(*k*) is the Fourier-space structure factor and Ω is the set of modes that captures the bundling transition (Fig. S2). Error bars are standard deviations from *N* = 5 images per concentration. Shaded regions demarcate concentrations above the bulk phase boundaries. Zoomed in TIRFM images of **e.** BuGZ and **f.** TPX2 bundles both at 2000 nM displaying film and droplet-like structures expected for filaments held together by capillary forces. Scale bars are 1 *μ*m.

Next, we mixed short (1–5 *μ*m in length, 25 nm in diameter), stabilized, ATTO647-labelled GMPCPP microtubules with either BuGZ or TPX2 at different concentrations and flowed the mixture into a flow chamber with the coverslip side down. After a 10 min incubation, bundles settled to the coverslip surface and were imaged with TIRFM. For BuGZ, robust microtubule bundles were only observed near and above the bulk phase boundary (Fig. 1a). For TPX2, microtubule bundling began between 12.5–25 nM (Fig. 1b). We quantified bundle formation with an order parameter defined as $\frac{\int_{\Omega }\;\;S(k)dk}{\int_{-\infty }^{\infty }\;\;S(k)dk}$ where *S*(*k*) is the Fourier-space structure factor of the TIRFM images and Ω is a set of modes that captures the bundling transition (Fig. 1c–d, Fig. S2, Supplementary Methods). For BuGZ, the bundling transition is clearly sigmoidal, consistent with dynamics driven by phase separation (Fig. 1c). The transition occurs just before the bulk phase boundary, likely due to prewetting effects. For TPX2, the bundling transition occurs over a larger concentration range, which may be attributed to TPX2’s ability to crosslink microtubules at the single-molecule level [49] as well as bundle them through condensation (Fig. 1d). For both proteins, we note the high background signal of soluble tubulin below the phase boundaries. Above the phase boundary, soluble tubulin gets sequestered into condensed MAPs as previously reported [20, 21], increasing the intensity contrast of the microtubule channel.

Liquid-like features, such as droplets and films, on microtubule bundles that are larger than the diffraction limit are visible for both BuGZ (Fig. 1e) and TPX2 (Fig. 1f) at sufficiently high MAP concentrations, since an increase in bulk MAP concentration above the phase boundary serves to increase the volume of the condensed phase [43]. Furthermore, decreasing the microtubule density in the assay by a factor of 10 resulted in smaller bundles for a given MAP concentration, but larger droplets along those bundles (Fig. S3). This observation is a consequence of more volume of condensed MAP per available microtubule surface area, resulting in bigger droplets along bundles [43]. A negative control in the absence of MAPs ruled out microtubule bundling from other forces such as depletion interactions [51] under our experimental conditions (Fig. S4). Such liquid-like features share striking resemblance to the micrometer-to-millimeter scale wetted fiber networks that are known to form bundles through capillary forces [39, 40].

As another control, we performed bundling experiments using Eg5 at low ATP concentrations (1 *μ*M), where it can still bind and crosslink microtubules but not slide them apart. We first confirmed that “static Eg5” does not phase separate (Fig. S5a), and thus can serve as a model of a bundler that acts solely by single-molecule crosslinking. In the bundling assay, bundles started to settle on the coverslip at concentrations above 2.5 nM Eg5 (Fig. S5b). This is an order of magnitude lower than BuGZ (above 50 nM), which can only bundle microtubules by condensation, and is comparable to TPX2 (above 5 nM), which can bundle microtubules via both mechanisms. No droplet or film structures were visible for static Eg5 bundles at any concentration. Furthermore, we do not observe any sequestering of soluble tubulin into regions of high Eg5 intensity, like we did for the condensed MAPs. Taken together, these observations motivated us to quantitatively investigate whether capillary forces are the dominant bundling mechanism for condensed MAPs.

### B. Direct measurement of condensate-driven capillary forces on microtubules

**FIG. 2.**
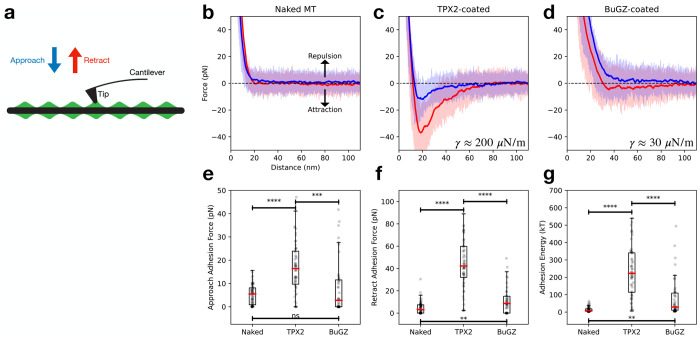
AFM measurements quantify interfacial forces. **a.** Schematic of experimental in-fluid AFM configuration. First, a microtubule is found using an AFM probe of tip radius *R_T_* ≈ 20 nm. A series of approach/retract force spectroscopy curves are then instantiated along the coated microtubule. Average approach (blue) and retract (red) curves for **b.** a naked microtubule (*N* = 65), **c.** a TPX2-coated microtubule (*N* = 54), and **d.** a BuGZ-coated microtubule (*N* = 46). The shaded blue/red regions correspond to the 25th and 75th percentile of approach/retraction force curves. Statistics of the **e.** approach adhesion force, **f.** retract adhesion force, and **g.** adhesion energy for naked, TPX2-coated, and BuGZ-coated microtubules. The red line is the median. Interfacial tensions for condensed TPX2 and BuGZ were estimated using the formula $F_{a}∼4\pi \gamma \overset{‾}{R}$, where *F_a_* is the average retract adhesion force and $\overset{‾}{R}=\frac{\sqrt{R_{T}}}{\sqrt{R_{T}^{-1}+R^{-1}}}$ where *R_T_* ≈ 20 nm is the nominal AFM tip radius and *R* ≈ 13 nm is the microtubule radius.

We sought to directly measure the magnitude of these capillary forces on microtubule surfaces by using in-fluid AFM. GMPCPP stabilized microtubules were electrostatically adhered to an atomically smooth mica surface and either TPX2 or BuGZ was added to a final concentration above their bulk phase boundary value (Fig. 2a). Microtubules coated with condensed MAP were first located using fast-tapping-mode AFM, after which we performed a series of slow approach-retract force ramps at specified locations along the lengths of microtubules (Supplementary Methods). The average force curves (Fig. 2c–d) exhibit significant adhesion compared to measurements of naked microtubules in the same buffer (Fig. 2b), both in the retract and approach ramps, where the magnitude of the adhesive force is the minimum of the force curve (Figs. 2e–f). We interpret these adhesive forces as condensate-mediated capillary bridges between the microtubule and the AFM silicon-nitride tip, both of which are negatively charged in our experimental conditions [52].

Using the minima of the averaged retract curve (Fig. 2c–d) as a measure for the adhesive force *F_a_*, we can estimate the interfacial tension of each condensate via $F_{a}∼4\pi \gamma \overset{‾}{R}$, where the effective radius is $\overset{‾}{R}=\frac{\sqrt{R_{T}}}{\sqrt{R_{T}^{-1}+R^{-1}}}$ (Supplementary Theory). This force expression assumes a highly idealized contact configuration of the capillary bridge extent being small relative to the AFM tip radius and microtubule radius, yet it can be used as an estimate for calculating the surface tension. *R_T_* ≈ 20 nm is the nominal AFM tip radius and *R* ≈ 13 nm is the microtubule radius. We find *γ*_TPX2_ ≈ 200 *μ*N/m and *γ*_BuGZ_ ≈ 30 *μ*N/m. These interfacial tension values measured with a nanoscale probe fall in the higher end of the range of previous measurements of condensate interfacial tensions that relied on other techniques that used micronscale probes [53]. Indeed, there are subtle differences arising at the nanoscale, such as the relative importance of the interface thickness [44], that could influence the effective measured value of γ. By measuring these forces at the nanoscale (Fig. 2b–f), we obtain a direct estimate of the relevant forces protein complexes and intracellular surfaces would experience from a single capillary bridge on microtubules.

To explain the order of magnitude difference in interfacial tensions between condensed TPX2 and BuGZ, we start with a Flory–Huggins-type phase field model [54–56] (Supplementary Theory). We find that the interfacial tension scales like

(1) $$\gamma =\frac{6k_{B}T\ell {\left(\chi -\chi_{c}\right)}^{3/2}}{\nu N^{1/2}\chi_{c}^{2}}$$

near the critical point, where *χ* is the mean-field interaction parameter, $\chi_{c}\approx \frac{1}{2}$, *N* is the degree of polymerization, and *l* and *ν* are the length and volume, respectively, that characterize the lattice size in the theory. The surface tension expression in Eq. (1) is derived only for regions in the phase diagram near the critical point, and may only be used approximately when relating the protein characteristics to their condensed phase surface tension more generally. Because *N* is similar between TPX2 and BuGZ, the difference in *γ* likely arises due to a difference in molecular interaction strength *χ* that drives phase separation. If we assume such interactions are dominated by electrostatics, a modified Voorn–Overbeek theory [57–59] gives $∼f^{2}$, where *f* is the fraction of charged amino acids in the protein chain (Supplementary Theory). We note that the Voorn–Overbeek theory applied here accounts for the effective electrostatic free energy of both positive and negatively charged sites along the polyampholytic protein backbone. While the predictions are limited by standard mean-field assumptions, the model can provide a useful estimate of the strength of the electrostatic interactions driving phase separation. We estimate *f*_TPX2_ ≈ 0.33 and *f*_BuGZ_ ≈ 0.20, which leads to *χ*_TPX2_ ≈ 2.3 and *χ*_BuGZ_ ≈ 0.9. This results in a prediction of $\frac{\gamma_{TPX2}}{\gamma_{BuGZ}}=O(10)$, which rationalizes the measured difference in *γ*. Thus, our theory connects sequence charge features of proteins to the interfacial tension of their condensed phase.

The AFM force curves can be integrated to generate energy curves, the minimum of which is the capillary adhesion energy *E_a_*. We measure adhesion energies per unit area $\frac{E_{a}}{\pi {\overset{‾}{R}}^{2}}$ in the range of $0.1-1\frac{k_{B}T}{{\;nm}^{2}}$ (Fig. 2g). We note that these adhesion energies are at least an order of magnitude higher than those measured for tubulin dimers on the interfaces of stress granules [44]. While this might reflect that condensed MAPs offer stronger adhesion specifically to tubulin lattices, we caution that comparing *in vitro* material properties to those *in vivo* is a qualitative endeavor, since condensates can be in different parts of their phase diagrams depending on environmental context, which affects the partition coefficient and therefore the interfacial tension [60].

In addition, we leveraged our AFM technique to measure the viscosities *μ* of the condensates (Fig. S6). To do so, we let bulk MAP droplets (sizes ≳ 1 *μ*m) sediment to the glass bottom of a dish and performed a force ramp with a larger 1 *μ*m nominal tip radius (Supplementary Methods). Using a lubrication analysis, we find average values of *μ*_TPX2_ ≈ *μ*_BuGZ_ ≈ 500 mPa·s for the condensed MAPs and *μ*_s_ ≈ 1 mPa·s for the assay buffer. We note that the condensates get more viscous over the timescale of minutes as they age, in agreement with previous observations (Fig. S6f-g) [61].

### C. Snapping dynamics of capillary bundles

**FIG. 3.**
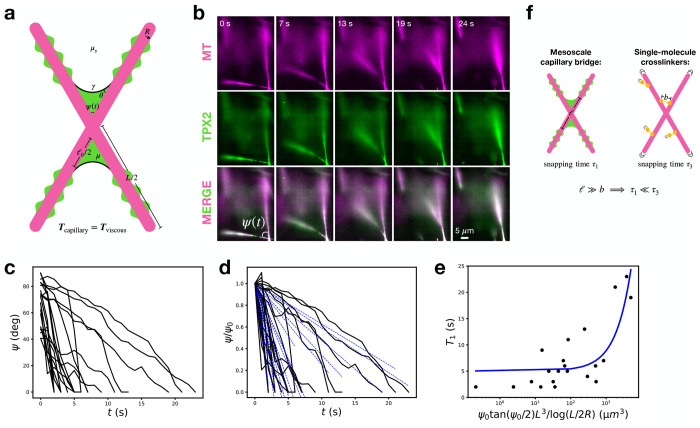
Snapping dynamics of capillary bundles. **a.** Schematic showing the non-equilibrium configuration of two microtubules of length *L* that contact at an arbitrary angle *ψ*(*t*). The microtubules are wetted by a condensate with surface tension *γ*, viscosity *μ*, and contact angle *θ* in a solvent of viscosity *μ*_s_. **b.** Oblique TIRFM live imaging of two bulk microtubule bundles coated with condensed TPX2 above the phase boundary (500 nM) snapping together. Background subtraction was done to enhance visual contrast. Scale bar is 5 *μ*m. **c.** Plot of oblique TIRFM measurements of snapping angles versus time. *N* = 21 snapping events. All data is taken with TPX2 at 500 nM. **d.** Plot of snapping angles versus time overlaying least-squares fits to Eq. (2) (dashed blue lines). **e.** Plot of snapping times versus the length of the shortest microtubule in the pair. The blue curve is the least-squares fit to the function $T_{1}=\left(\frac{\psi_{0}}{2}\right)tan\left(\frac{\psi_{0}}{2}\right)\tau_{1}$, where $\tau_{1}=\frac{\pi \mu_{s}}{6\gamma R\ell_{0}}\left(L^{3}/log(L/2R)\right)$. All parameters are independently measured or known except $\ell_{0}$, which we use as a least-squares fitting parameter, finding $\ell_{0}=9\pm 1\;nm$. **f.** Schematic contrasting capillary bundling versus single-molecule crosslinking. Because the wetted length *ℓ* is a mesoscale length scale that can approach the microtubule length *L* for late snapping times, we expect capillary torques to be generically larger and snap microtubules more quickly than crosslinking torques, whose moment arm is limited by a molecular length scale *b*.

To rationalize the dynamics of bundle formation, we developed a theoretical model where two microtubules contact each other at an arbitrary initial angle *ψ*_0_ that evolves in time as ψ(t) via a liquid capillary bridge with contact angle *θ* (Fig. 3a). There is both an adhesive capillary force [62] that acts to bring the microtubules closer together and a capillary torque [63, 64] that acts to drive the liquid-liquid interface to a state of minimal energy as well as to maximize the contact the condensate has with the microtubules. We assume that the condensate interface relaxes more quickly than the rate of microtubule motion, so that it is always locally equilibrated (Supplementary Theory). This predicts an equilibrium state of bundled microtubules that are parallel and wetted by the condensate.

For early times ($\psi \to \psi_{0}$) the capillary torque is primarily resisted by dragging the filaments together through the solvent of viscosity *μ_s_*. Assuming perfect wetting (*θ* = 0) we find that the angle ψ should decrease linearly in time as

(2) $$\psi (t)∼\psi_{0}-\frac{2}{tan\left(\psi_{0}/2\right)\tau_{1}}t,$$

where $\tau_{1}=\frac{\mu_{s}L^{3}}{6\gamma R\ell_{0}log(L/2R)}$, *L* is the microtubule length, *R* is the microtubule radius, *γ* is the interfacial tension, and *l*_0_ is the initial wetted length of the droplet that contacts both filaments.

To test these ideas, we mixed short, stabilized, ATTO647-labelled GMPCPP microtubules with 500 nM TPX2 in a well and imaged bundle formation live in 3D using oblique TIRFM (Supplementary Methods). We directly observed TPX2-coated microtubules contacting at an initial angle and snapping together until they formed a parallel bundle, as predicted (Fig. 3b, Fig. S7). We also quantified individual snapping trajectories versus time (Fig. 3c–d), finding linear behavior at our experimentally observable temporal resolution, validating Eq. (2).

Using Eq. (2), we can compute the time it takes microtubules to fully snap together as $\psi \left(T_{1}\right)=0⟹T_{1}=\left(\frac{\psi_{0}}{2}\right)tan\left(\frac{\psi_{0}}{2}\right)\tau_{1}$. We fit this model to our data, where the only fit parameter is *l*_0_, since we measured *μ*_s_ and *γ* using AFM and take *R* = 12.5 nm. The model captures the data reasonably well across our entire range of measured *L* and $\psi_{0}$ (Fig. 3e), where for *L* we take the smallest microtubule length in the pair. From the fitting we learn $\ell_{0}=9\pm 1\;nm$, which is on the order of *R*, which is geometrically sensible for the initial wetted length upon microtubule contact.

How does this mechanism of microtubule bundling via capillary bridges fundamentally differ from bundling via single-molecule crosslinkers? During the late time (ψ→0) dynamics of capillary snapping, the wetted length increases significantly as $\ell ∼\ell_{0}\sqrt{\frac{\psi_{0}}{\psi }}$ and the angle decreases as $\psi ∼{\left(\frac{\tau_{2}}{t}\right)}^{2/5}$ (Supplementary Theory). In this regime, the squeezing of the intervening condensate dominates the viscous torque at shallow angles. The governing time scale $\tau_{2}=\frac{\mu \ell_{0}\sqrt{\psi_{0}}}{20\gamma }$ results from the balance of the capillary torque and the viscous resistance from squeezing the condensed film of viscosity *μ* between aligning filaments. Measuring these late time dynamics offer a way of probing a feature unique to capillary bridges. However, for the parameters we measure $\tau_{2}=O\left(10^{-5}\right)s$, which is too fast to observe using our current experimental set up.

In standard models of microtubule bundling by singlemolecule crosslinkers, the crosslinkers are modeled as molecular springs of a characteristic length *b* that can bind two microtubules [7, 8]. Such a model predicts a characteristic time scale $\tau_{3}=\frac{\pi \mu_{s}L^{3}}{qkb^{3}log(L/2R)}$ , where *k* is the spring constant and *q* is the crosslinker density per unit microtubule length (Supplementary Theory). If we assume similar adhesion force scales between the two models so kb∼γR, and we take qb∼1, we find that the ratio of timescales is given by $\frac{\tau_{1}}{\tau_{3}}∼\frac{b}{6\pi \ell }$. Because *b* is a molecular length scale and ℓ is the mesoscopic length scale of the wetted film, we expect *τ*1 ≪ *τ*3 (Fig. 3f). That is, even for comparable adhesion forces, we generally expect capillary torques to be at least an order of magnitude larger than crosslinking torques due to their mesoscale nature. For example, in the study of synthetic microtubules crosslinkers by Drechsler et al. [11], it takes their microtubules ≈ 6 min to snap together, whereas all our snapping events occur in less than 30 s, which supports our bundling argument.

### D. Sliding dynamics of capillary bundles

In addition to the adhesive force that bundles microtubules together, we predict that capillary bridges should provide *viscous* resistance to intrabundle microtubule sliding. Relative microtubule sliding, usually driven by molecular motors, is prevalent in nature with examples ranging from mitotic spindle maintenance [65, 66] to paradigmatic active matter models [67]. Understanding the resistance provided to motor-driven sliding by condensate-mediated capillary bridges is therefore an important biophysical question, which will additionally yield further insights into how capillary bundles differ from microtubules bundled by single-molecule crosslinkers.

**FIG. 4.**
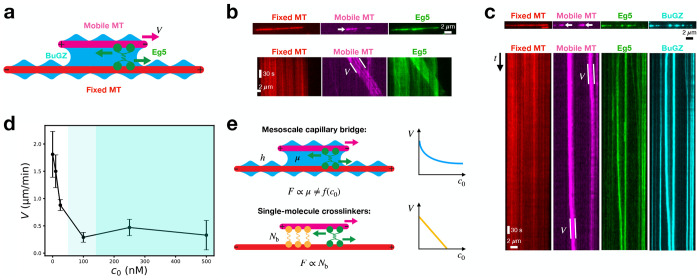
Sliding dynamics of capillary bundles. **a.** Schematic of the 4-color TIRFM experimental set up. Long, Alexa568-labeled biotinylated microtubules are strongly adhered to a passivated coverslip functionalized with neutravidin. Eg5 binds to these long microtubules and forms sandwiches with short, mobile ATTO647-labeled microtubules. If microtubules in the sandwich are antiparallel, Eg5 will slide the short, mobile microtubule towards its minus-end. The experiment is done as a function of BuGZ concentration below and above the bulk phase boundary. Snapshot and kymograph of a **b.** 0 nM BuGZ and 100 nM Eg5 experiment and a **c.** 500 nM BuGZ and 100 nM Eg5 experiment. The velocity *V* of the short, mobile microtubule is computed from the slope of the mobile microtubule kymograph. Scale bars are 2 *μ*m and 30 sec. **d.** Behavior of the sliding velocity *V* of the short, mobile microtubule as a function of the concentration of BuGZ *c*_0_ (black line). Error bars are standard deviations. *N* > 20 microtubules per condition. The darker shaded blue region represents BuGZ concentrations above the bulk phase boundary while the lighter shaded blue region is an estimate for the prewetting zone for BuGZ condensation based on the bundling transition in Fig. 1e. Below the phase boundary, *V* decreases with *c*_0_, whereas above the phase boundary, a viscous capillary bridge is established whose sliding resistance is insensitive to *c*_0_. Orange dashed line shows the predicted behavior if sliding resistance was due to single molecule cross-linkers that bind strongly enough to stall Eg5 sliding. **e.** Schematic comparing how single-molecule crosslinkers versus capillary bridges resist microtubule sliding. Crosslinkers bind and resist sliding in a manner proportional to the number of bound crosslinkers *N_b_* until microtubules stall [70]. Near and above the phase boundary, condensates with a viscosity *μ* wet microtubules providing viscous resistance to sliding. Any further increase in bulk MAP concentration does not change the concentration of the dense phase nor its viscosity, and hence sliding resistance can saturate below the stall force.

To assess this, we performed *in vitro* sliding assays using purified recombinant Eg5-GFP and monitored how motor-driven sliding velocities changed as we titrated in BuGZ from nanomolar concentrations to above the phase boundary (Fig. 4a). We focused on BuGZ to avoid a known binding interaction between TPX2 and Eg5 [68]. We first flowed in long (> 5 *μ*m) Alexa568-labeled, biotinylated GMPCPP microtubules into a flow chamber with a passivated and neutravidin-functionalized coverslip. We then serially flowed in 100 nM of Eg5, short ATTO647-labelled GMPCPP microtubules, and a final mixture of 100 nM Eg5 and the final bulk concentration *c*_0_ of BuGZ. The reaction was imaged using TIRFM and the Eg5-driven sliding speeds *V* of the short microtubules were measured by constructing kymographs for each mobile microtubule. In the absence of BuGZ (*c*_0_ = 0), we measured *V* = 1.8 ± 0.4 *μ*m/min for Eg5-driven sliding, in excellent agreement with previous measurements (Fig. 4b) [69]. As we increased the concentration *c*_0_ of BuGZ, we observed two regimes of behavior. Below the phase boundary and prewetting transition (as defined by the onset of microtubule bundling in Fig. 1c), the sliding speed *V* decreased with increasing *c*_0_ . However, above the prewetting transition we observe a saturation of *V* to a constant value with no further dependence on *c*_0_ (Fig. 4c–d, Fig. S8).

We rationalize these observations as follows. Below the phase boundary, BuGZ binds the microtubule lattice at the single-molecule level and offers discrete roadblocks that can impair Eg5 sliding processivity (Fig. S8a-b). As *c*_0_ increases, more BuGZ binds, increasing the density of roadblocks and thus decreasing *V* . Near and above the phase boundary, condensed BuGZ fully wets the entire microtubule lattice and forms a capillary bridge with the mobile microtubule (Fig. 4c, Fig. S8c-d). In this scenario, the main resistance to sliding comes from the viscosity of the dense phase which scales like $F∼\frac{\mu V}{h}A_{\text{wet}\;}$, where *h* is the distance between microtubules and *A_wet_* is the total area of microtubule lattice wetted by the capillary bridge. Since both microtubules are already fully wetted at this point, any further increase in *c*_0_ serves only to change the volume and shape of the bridge [62], which has a sub-dominant effect on *F* , as the majority of viscous shearing happens in the gap between microtubules. The crucial point here is that the condensate viscosity *μ* dominates the resistance, which is not a function of *c*_0_ above the phase boundary since the dense phase concentration remains constant (Fig. 4e).

How can we determine whether our measurements of condensed BuGZ viscosity are consistent with the decreased sliding velocity we observe in Fig. 4d? Using a simple model (Supplementary Theory), we find that the ratio of sliding velocities $V_{f}/V_{s}$, where *V_s_* is the velocity with just buffer (viscosity *μ_s_* ) between the microtubules and *V_f_* is the velocity with an additional condensed film (viscosity *μ*) of thickness *h* between the microtubules, is

(3) $$\frac{V_{f}}{V_{s}}=\frac{1}{1+\frac{\mu Rlog\left(\frac{L}{2R}\right)}{2\mu_{s}h}}$$

Keeping discussion at the level of order-of-magnitude estimates due to the measured spread and time-dependence of the viscosity (Fig. S6f-g), we have $\frac{\mu }{\mu_{s}}=O\left(10^{2}\right)$, $log\left(\frac{L}{2R}\right)=O(1)$, and $\frac{R}{h}\approx 0.2$, where we take *h* = 60 nm, which is the length of Eg5 [69]. This gives *V_f_*/*V_s_* ≈ 0.1 which compares well to our measured value of 0.2, which we compute from Fig. 4d by averaging the velocities for 100–500 nM BuGZ and dividing it by the velocity for 0 nM BuGZ.

An alternative hypothesis is that bulk BuGZ condensates could sequester Eg5 into an inactive state, lowering the active population on the microtubule lattice and hence potentially lowering *V*. We ruled this out by performing sliding experiments at lower concentrations of Eg5, showing that *V* is insensitive to decreasing Eg5 concentrations over an order of magnitude (Fig. S9a-d). We also confirmed that while Eg5 density along microtubules measurably decreases upon first addition of BuGZ, it remains the same for all higher BuGZ concentrations (Fig. S9e). Taken together, the picture of constant Eg5 forcing across all BuGZ concentrations is reasonable, and thus we can attribute the decrease in sliding speed predominately to viscous resistance imparted by BuGZ.

This viscous resistance picture differs significantly from the sliding resistance imparted by crosslinkers that can individually bundle microtubules at the single-molecule level (Fig. 4e). For example, a similar assay [10] showed that the Eg5-driven sliding velocity *V* decreased in the presence of the homodimeric crosslinker PRC1 from 1.4±0.4 *μ*m/min at [PRC1] = 0.034 nM to 0.58±0.18 *μ*m/min at [PRC1] = 0.54 nM. This ∼ 60% decrease in *V* even at < 1 nM concentrations is in a totally different regime than our data (Fig. 4d), and reflects the fact that single-molecule crosslinkers provide sliding resistance at far lower concentrations than condensed MAPs that only bundle microtubules through viscous capillary bridges. Separate measurements using optical tweezers found that PRC1 crosslinkers resisted microtubule sliding in a manner directly proportional to the number of bound PRC1 crosslinkers [70], eventually resulting in microtubule stalling, which is different from the nonlinear response we measure here. Therefore, viscous capillary bridges can provide a way of generating resistance to motor-driven microtubule sliding that is below the motor stall force and insensitive to the bulk bundler concentration.

### III. DISCUSSION

In this work, we established that condensed MAPs can bundle microtubules through capillary forces. Using BuGZ and TPX2 as model MAPs, we observed robust microtubule bundling *in vitro* with droplet and film-like structures reminiscent of liquid capillary bridges optically visible above the bulk phase boundary. Using AFM, we directly measured the interfacial tension and viscosity of condensed MAPs, and derived a theory for how the interfacial tension depends on amino acid sequence charge. We then measured the snapping dynamics of live bundle formation and found agreement with a theoretical model of a capillary bridge snapping filaments together via the interfacial tension of the condensate. Lastly, we showed how Eg5-driven microtubule sliding is resisted by the presence of a condensed MAP, finding that above the phase boundary the resistance is insensitive to the MAP concentration. We hypothesize this is due to capillary bridges providing purely viscous resistance to sliding, in which the condensate viscosity is insensitive to the MAP concentration. Taken together, we presented a novel microtubule bundling mechanism, which is distinct from the previously established way of bundling via single molecule crosslinkers with at least two microtubule binding regions. Our results have several implications, which we discuss below.

First, *in vitro* observations of microtubule bundling by MAPs do not imply that the MAP can crosslink microtubules at the single-molecule level via two microtubule binding regions. A simple bulk phase separation assay that tests whether the MAP in question forms condensates at the experimental conditions of interest should be done before making any conclusions about the underlying microtubule bundling mechanism.

We stress that our model of microtubule bundling by capillary forces is insensitive to the molecular details that give rise to the condensed phase. The molecular nature of the interactions that give rise to condensates is an issue that is still debated [71, 72]. However, irrespective of the molecular mechanism of condensate biogenesis, as long as such a mesoscale compartment *exists* with internal fluidlike turnover kinetics and affinity for the microtubule lattice, capillary forces can be the dominant mechanism of microtubule bundling. Moreover, our usage of BuGZ and TPX2 in this work was purely as model proteins to study the biophysical aspects of capillary bundling. We do not claim that this mechanism is at play in living cells for these proteins, as that is likely context dependent and would require additional direct investigation [16].

While capillary bundling is a general mechanism that can explain how condensed MAPs bundle microtubules, other effects could be at play that do not rely on interfacial tension, especially between the prewetting transition and the bulk phase boundary. For example, the mechanism of tau-mediated microtubule bundling is complex and still debated, with hypotheses ranging from transient electrostatic dimers [73] to a viscoelastic network of crosslinked tau-tubulin oligomers [74]. While it is clear that condensed tau can form capillary bridges between microtubules under certain conditions [24], it likely depends on tau concentration, ionic strength, and other physicochemical factors. Even for the model proteins studied here, future separation of function mutants that can bind microtubules but not form condensates, and vice versa, would be helpful in truly isolating the effects of capillary forces in bundling by eliminating potential cooperative binding effects that might contribute to the bundling dynamics.

Our model assumes the simplest condensate rheology: that of a viscous Newtonian fluid. While this is sufficient to capture the main interfacial driving force, it is known that condensates in general are viscoelastic with frequency-dependent material properties [61, 75, 76]. How such rheology affects the dynamics of interfacialtension-driven processes such as capillary bundling is an area ripe for future research.

Because all our experiments are done under well-mixed conditions, the entire microtubule surface is coated with condensed MAP above the phase boundary. This is not necessary for the capillary bundling mechanism to operate. Rather, a condensed droplet could nucleate locally along any part of the microtubule, which can then go on to form a capillary bridge with another microtubule, thus utilizing protein amounts much more efficiently. This approach has already been employed to apply local capillary forces to genetic loci using condensates [42]. In the other extreme, over-expression of MAPs that can condense could lead to uncontrolled and unregulated microtubule bundling, which has already been observed for CLIP-170 [28] and TPX2 [77]. This concept could help explain certain cancer cell phenotypes [78] or other deleterious cell states.

The concept of bundling via capillary forces has been recently applied to rationalize the bundling behavior of actin filaments in the presence of FUS [79]. We anticipate that this mechanism will be relevant for intermediate filaments and septins as well. More broadly, the regulation and misregulation of condensates applying capillary forces on cytoskeletal filaments represent a fascinating direction for future study at the intersection of molecular biology, soft matter physics, and disease physiology [48].

## Supplementary Material

1
