# Supplementary material for: Capillary bundling of microtubules by condensates
Source: bioRxiv. 2026 Jun 20:2026.06.19.733462. Preprint. [Version 1] doi: 10.64898/2026.06.19.733462 (PMC13308056; doi:10.64898/2026.06.19.733462)
Supplement: 1 [file NIHPP2026.06.19.733462v1-supplement-1.pdf]

bioRxiv preprint doi: https://doi.org/10.64898/2026.06.19.733462; this version posted June 20, 2026. The copyright holder for this preprint (which was not certified by peer review) is the author/funder, who has granted bioRxiv a license to display the preprint in perpetuity. It is made available under a CC-BY 4.0 International license.

SUPPLEMENTARY FIGURES

bioRxiv preprint doi: https://doi.org/10.64898/2026.06.19.733462; this version posted June 20, 2026. The copyright holder for this preprint (which was not certified by peer review) is the author/funder, who has granted bioRxiv a license to display the preprint in perpetuity. It is made available under a CC-BY 4.0 International license.

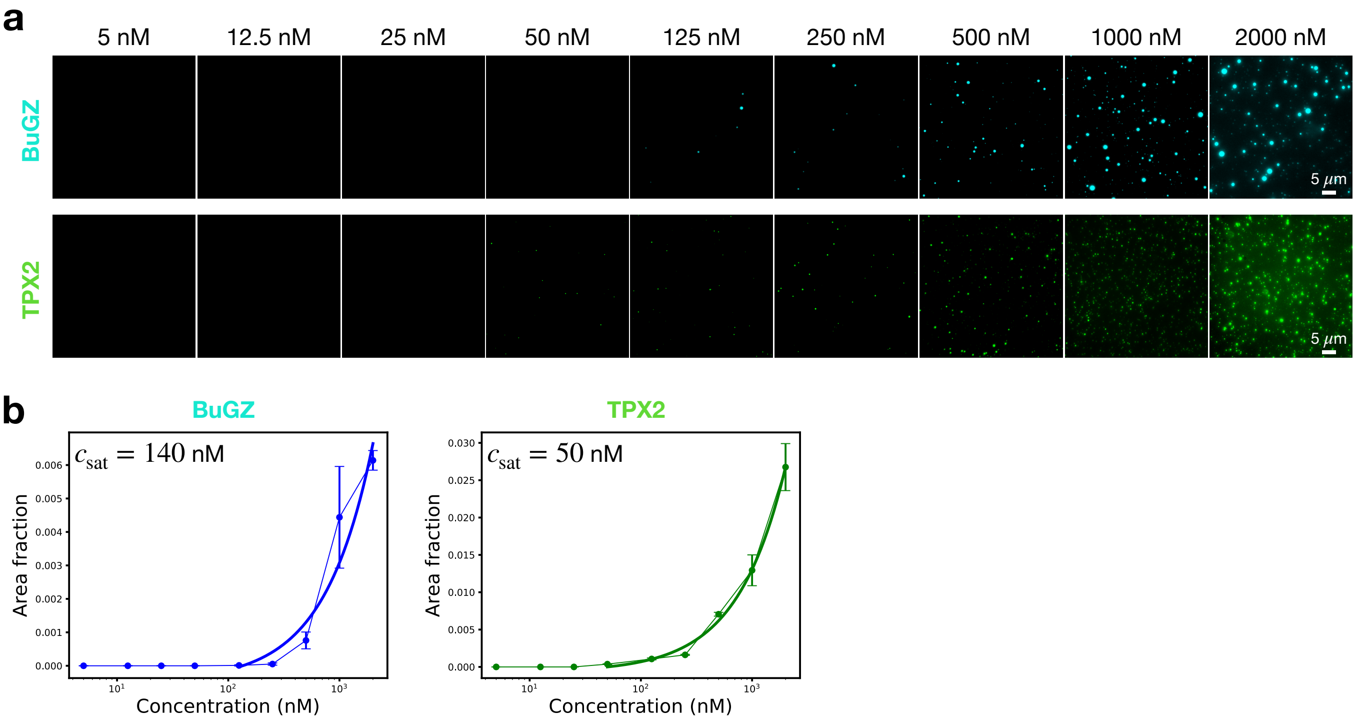

FIG. S1. **Bulk phase diagrams of BuGZ and TPX2. a.** Epifluorescence images of bulk phase separation assays for BuGZ and TPX2. Lookup tables are the same across concentrations for each protein to enable direct comparison. Scale bars are 5 μm. **b.** Phase diagrams for BuGZ and TPX2 showing area fraction of the condensed phase versus bulk concentration. The condensed phase was defined as regions having an intensity value above the threshold calculated using Otsu's method for the highest concentration images for each protein.

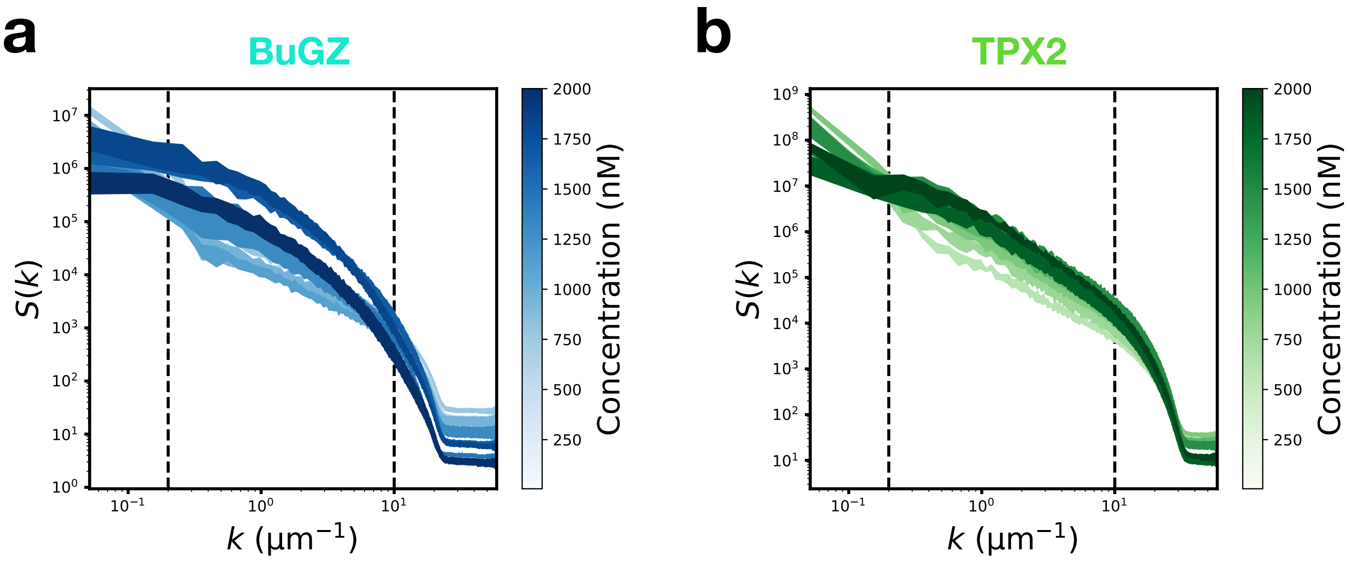

FIG. S2. **Structure factor of microtubule bundles.** Average structure factor $S(k)$ calculated from the microtubule intensity channels for **a.** BuGZ and **b.** TPX2 as a function of bulk MAP concentration. The set of modes Ω used to compute the order parameter in Fig. 1c-d is demarcated by the black vertical lines. Shaded error bars are standard deviations from $N = 5$ images per concentration.

bioRxiv preprint doi: https://doi.org/10.64898/2026.06.19.733462; this version posted June 20, 2026. The copyright holder for this preprint (which was not certified by peer review) is the author/funder, who has granted bioRxiv a license to display the preprint in perpetuity. It is made available under a CC-BY 4.0 International license.

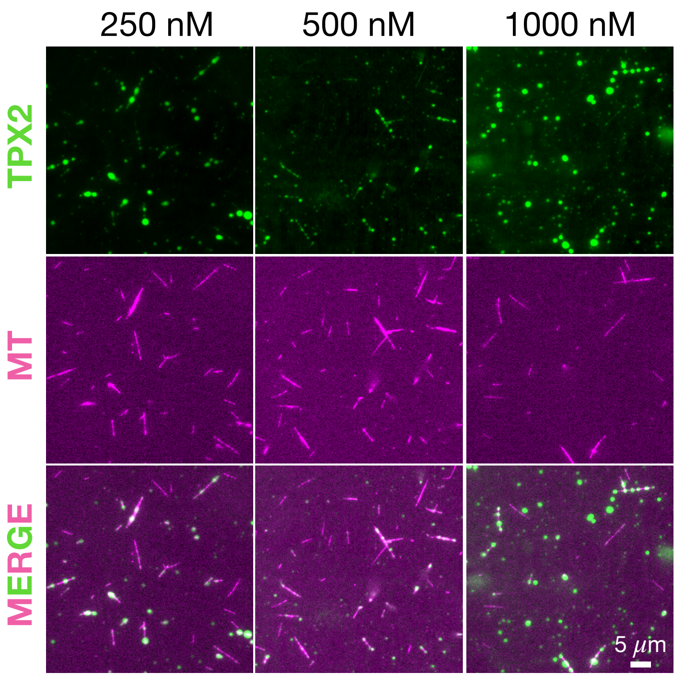

FIG. S3. **Structure of capillary bundles depend on microtubule density.** TIRFM images of microtubule bundles that sedimented to the flow channel surface after a 10 min incubation with TPX2. Experiments done at 1/10th the microtubule density relative to those in Fig. 1. At lower microtubule densities for a given TPX2 concentration, bundles are smaller in size while TPX2 droplets are bigger owing to there being more condensed TPX2 per surface area of microtubules. Scale bar is 5 μm.

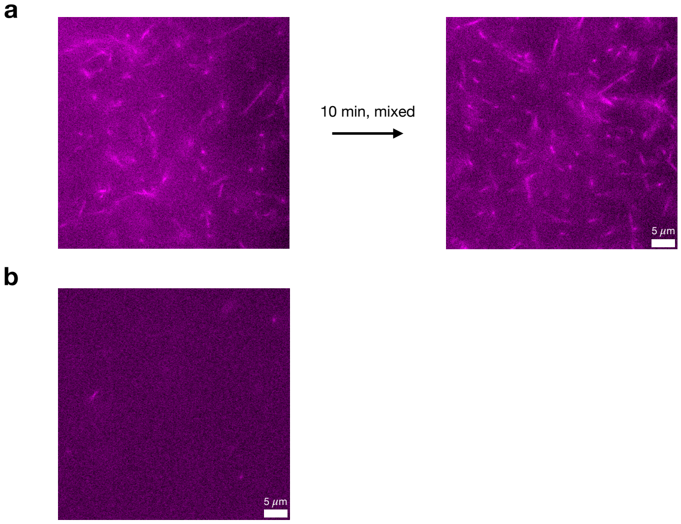

FIG. S4. **Negative control rules out depletion interaction for microtubule bundling. a.** Oblique TIRFM images of bulk microtubules before and after mixing in assay buffer without bundling MAPs. This shows that depletion forces alone do not bundle microtubules in our assay buffer and at our tested microtubule densities. **b.** TIRFM images at the surface 10 minutes after mixing show that no microtubule bundles sediment. Scale bars are 5 μm.

bioRxiv preprint doi: https://doi.org/10.64898/2026.06.19.733462; this version posted June 20, 2026. The copyright holder for this preprint (which was not certified by peer review) is the author/funder, who has granted bioRxiv a license to display the preprint in perpetuity. It is made available under a CC-BY 4.0 International license.

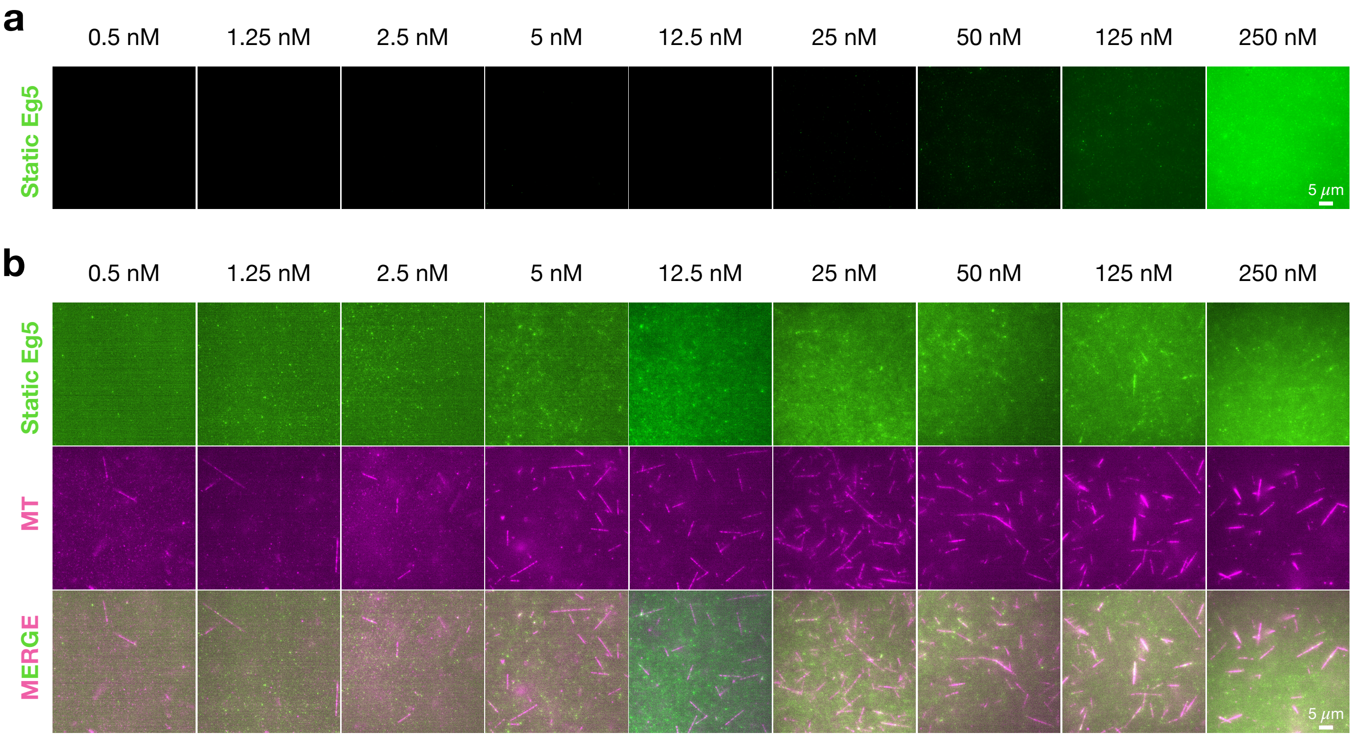

FIG. S5. **Static Eg5 bulk phase and bundling assay. a.** Epifluorescence images of bulk phase separation assays for Eg5 at low (1 μM) ATP concentrations ("static Eg5"). No robust mesoscale droplet formation is observed. Lookup tables are the same across concentrations to enable direct comparison. Scale bars are 5 μm. **b.** TIRFM images of microtubule bundles that sedimented to the flow channel surface after a 10 min incubation with static Eg5. Microtubule channel lookup tables are the same across concentrations to enable direct comparison. MAP channel look-up tables are optimized per concentration to allow for visualization. Scale bars are 5 μm.

bioRxiv preprint doi: https://doi.org/10.64898/2026.06.19.733462; this version posted June 20, 2026. The copyright holder for this preprint (which was not certified by peer review) is the author/funder, who has granted bioRxiv a license to display the preprint in perpetuity. It is made available under a CC-BY 4.0 International license.

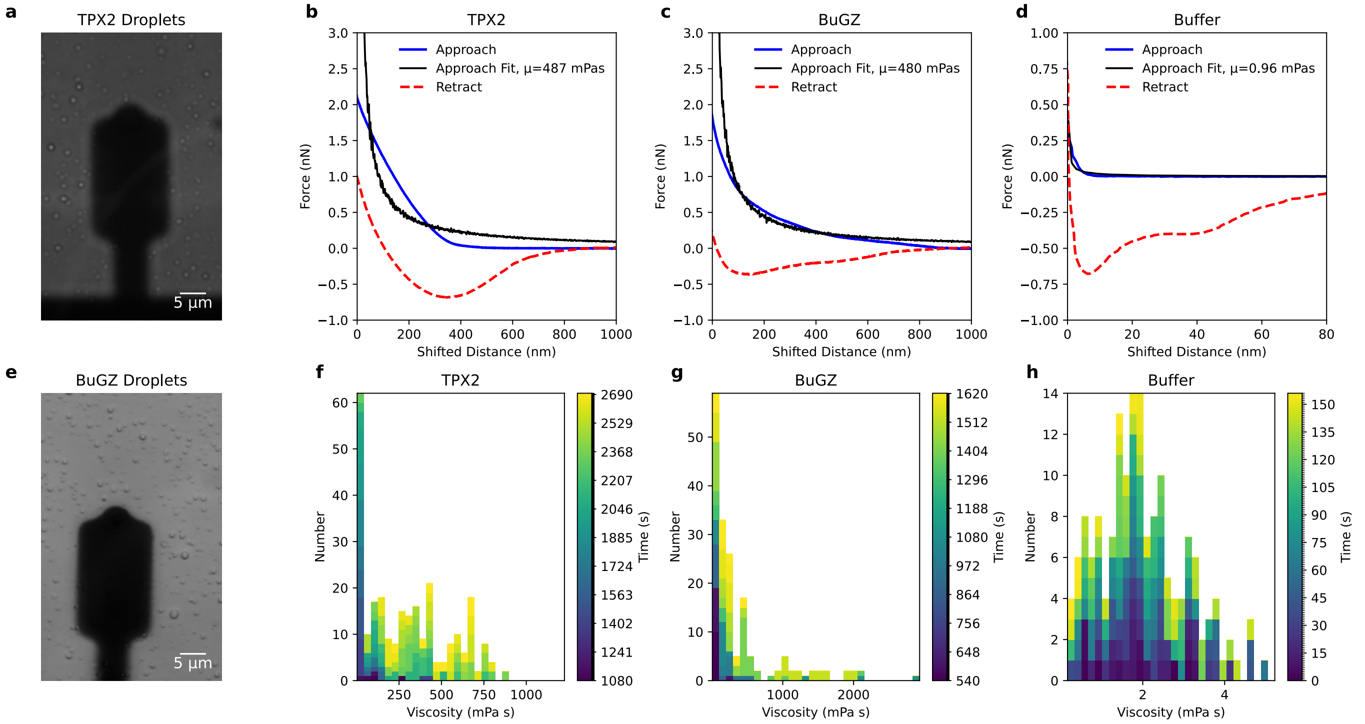

FIG. S6. **AFM measurements on bulk protein droplets.** Brightfield images of droplets below an AFM cantilever for **a.** TPX2 and **e.** BuGZ at 4 μM concentration. Average force curves for **b.** TPX2, **c.** BuGZ, and **d.** buffer including the approach (blue) and retract (red) curves. The shifted distance accounts for the cantilever deflection and estimates the position of the solid glass surface when it reaches zero. The black best fit curve fits the average force curve to the lubrication force prediction of a Newtonian fluid, taking into account the temporally measured average height profile and velocity. The approach fit is used to extract the viscosity, while the retraction minimum for TPX2 and BuGZ are used to estimate the surface tension of the bulk droplet (Supplementary Methods). The fitted viscosities are 487 mPa·s, 480 mPa·s, and 0.96 mPa·s, and the effective inferred surface tensions of the bulk droplets are 50 and 30 μN/m. Histograms of individual fitted viscosities for **f.** TPX2, **g.** BuGZ, and **h.** buffer, with outliers greater than three standard deviations excluded. The histograms include 267, 174, and 163 fitted viscosities from individual approach curves for TPX2, BuGZ, and buffer. The color bar indicates the time at which a given measurement was made after droplets first formed with an error of $\pm$60 s. The average viscosities from these histograms are 2.0, 303, 383 mPa·s, with standard deviations of 1.1, 257, and 546 mPa·s.

bioRxiv preprint doi: https://doi.org/10.64898/2026.06.19.733462; this version posted June 20, 2026. The copyright holder for this preprint (which was not certified by peer review) is the author/funder, who has granted bioRxiv a license to display the preprint in perpetuity. It is made available under a CC-BY 4.0 International license.

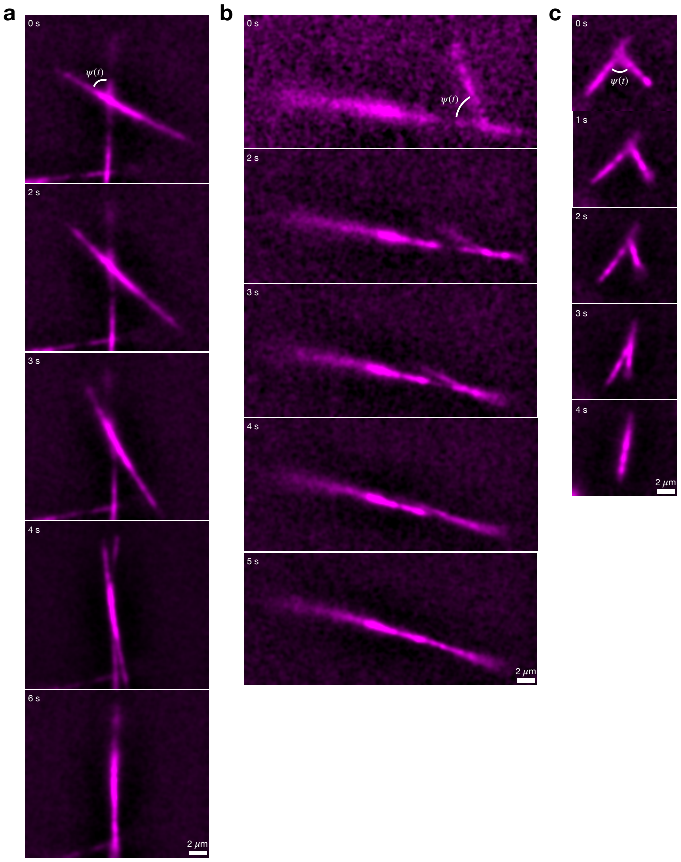

FIG. S7. **Additional examples of snapping dynamics.** Three additional examples of oblique TIRFM live imaging of two bulk microtubule bundles coated with condensed TPX2 above the phase boundary (500 nM) snapping together. Only the microtubule channel is shown. Images were bandpass filtered in Fourier space using a 23 μm lower cutoff and a 310 μm upper cutoff to maximize clarity for visualizing the snapping angle between microtubules. Scale bars are 2 μm.

bioRxiv preprint doi: https://doi.org/10.64898/2026.06.19.733462; this version posted June 20, 2026. The copyright holder for this preprint (which was not certified by peer review) is the author/funder, who has granted bioRxiv a license to display the preprint in perpetuity. It is made available under a CC-BY 4.0 International license.

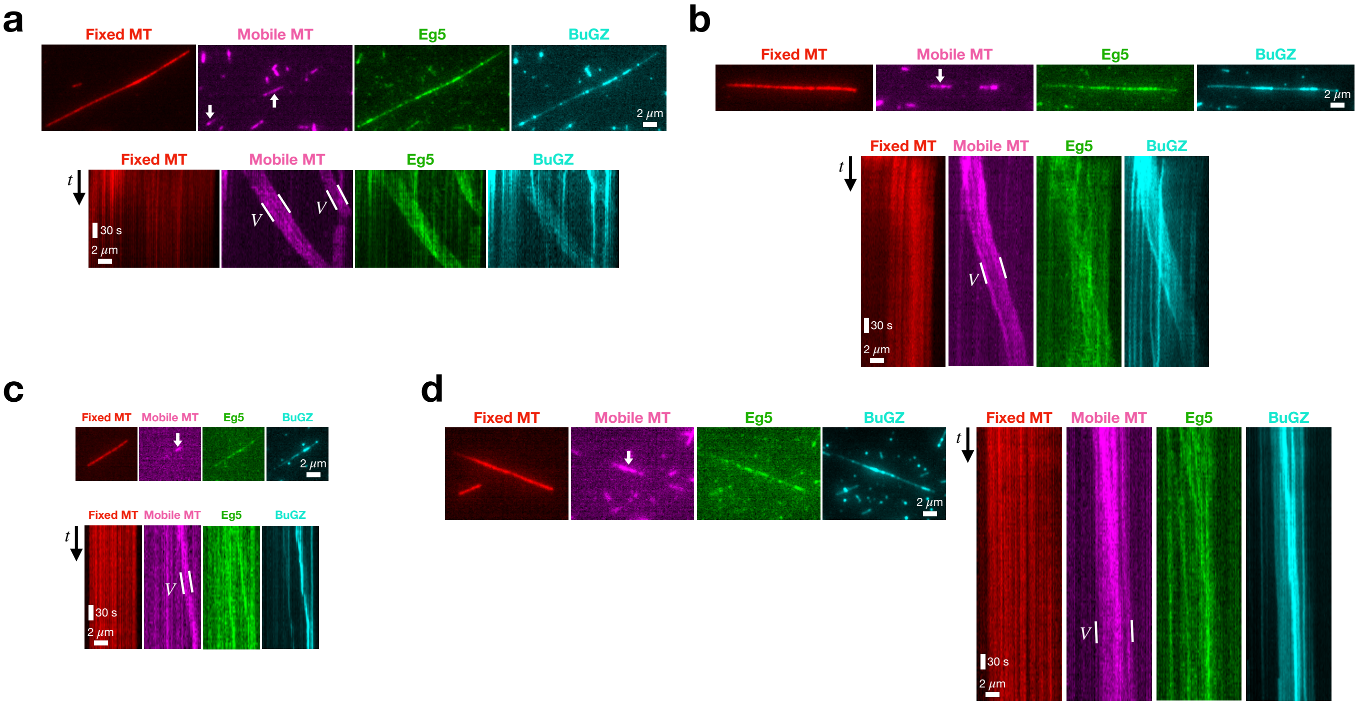

FIG. S8. **Additional sliding kymographs.** Snapshots and kymographs of **a.** 10 nM BuGZ, **b.** 25 nM BuGZ, **c.** 100 nM BuGZ, and **d.** 250 nM BuGZ sliding experiments all at 100 nM Eg5. The velocity V of the short, mobile microtubule is computed from the slope of the mobile microtubule kymograph. Scale bars are 2 μm and 30 sec.

bioRxiv preprint doi: https://doi.org/10.64898/2026.06.19.733462; this version posted June 20, 2026. The copyright holder for this preprint (which was not certified by peer review) is the author/funder, who has granted bioRxiv a license to display the preprint in perpetuity. It is made available under a CC-BY 4.0 International license.

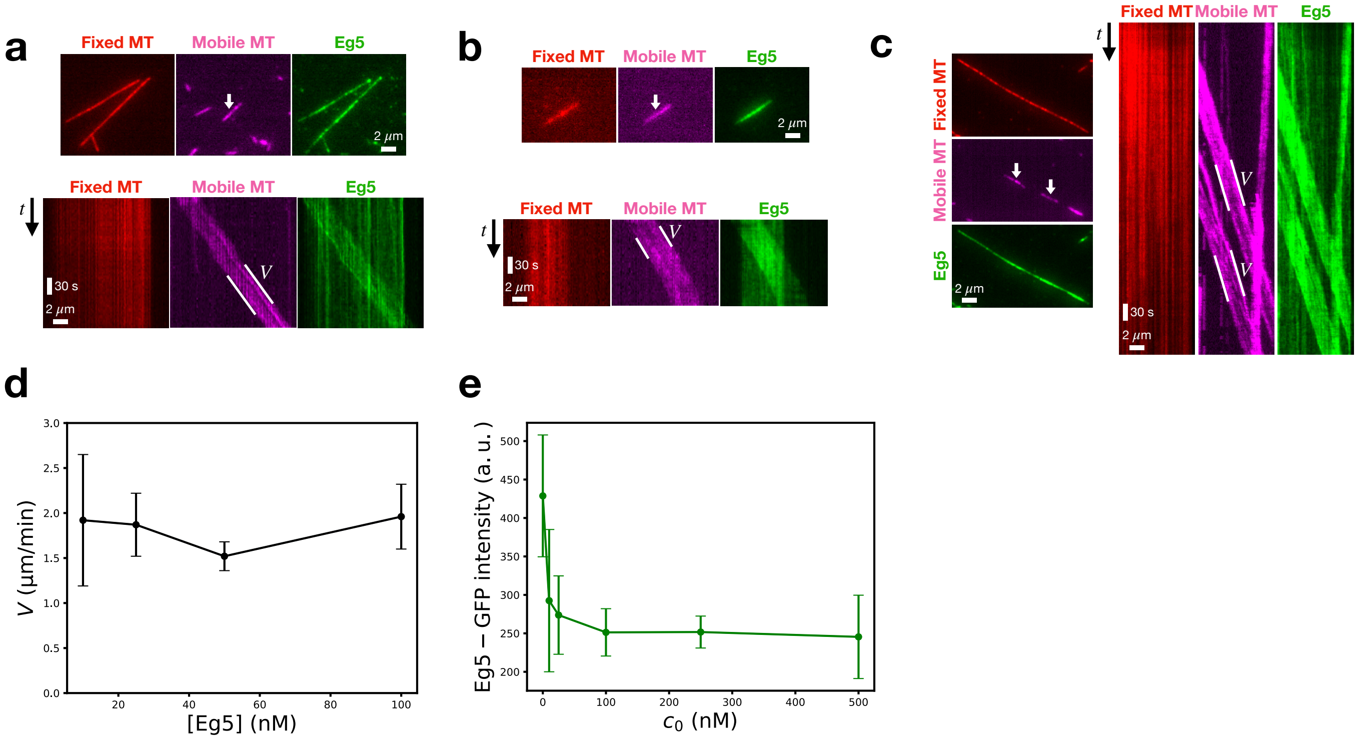

FIG. S9. **Lower concentration of Eg5 cannot explain observed decrease in sliding velocity.** Snapshots and kymographs of **a.** 10 nM Eg5, **b.** 25 nM Eg5, and **c.** 50 nM Eg5 in the absence of BuGZ. The velocity V of the short, mobile microtubule is computed from the slope of the mobile microtubule kymograph. Scale bars are 2 μm and 30 sec. **d.** Behavior of the sliding velocity V of the short, mobile microtubule as a function of only Eg5 concentration (black line), showing V does not measurably depend on the bulk Eg5 concentration. Error bars are standard deviations. $N > 20$ microtubules per condition. **e.** Eg5 fluorescence intensity on microtubules as a function of bulk BuGZ concentration. After measurably falling upon BuGZ addition, Eg5 concentration on microtubules remains roughly constant for all non-zero BuGZ concentrations. Error bars are standard deviations. $N > 20$ microtubules per condition.

bioRxiv preprint doi: https://doi.org/10.64898/2026.06.19.733462; this version posted June 20, 2026. The copyright holder for this preprint (which was not certified by peer review) is the author/funder, who has granted bioRxiv a license to display the preprint in perpetuity. It is made available under a CC-BY 4.0 International license.

MOVIE LEGENDS

Movie S1: Oblique TIRFM live imaging of two bulk microtubule bundles coated with condensed TPX2 above the phase boundary (500 nM) snapping together. Background subtraction was done to enhance visual contrast. TPX2 channel is in green; microtubule channel is in magenta. Scale bar is 5 μm. This movie corresponds to Fig. 3b.

Movie S2: Another example of oblique TIRFM live imaging of two bulk microtubule bundles coated with condensed TPX2 above the phase boundary (500 nM) snapping together. Only the microtubule channel is shown. Background subtraction was done to enhance visual contrast. Scale bar is 5 μm. This movie corresponds to Fig. S5a.

Movie S3: TIRFM live imaging of Eg5 (green) driven sliding of a mobile short microtubule (magenta) along an anchored long microtubule (red). Background subtraction was done to enhance visual contrast. Scale bar is 1 μm. This movie corresponds to Fig. 4b.

Movie S4: TIRFM live imaging of Eg5 (green channel) driven sliding of a mobile short microtubule (magenta) along an anchored long microtubule (red) in the presence of 100 nM BuGZ (cyan). Background subtraction was done to enhance visual contrast. Scale bar is 1 μm. This movie corresponds to Fig. S6c.

bioRxiv preprint doi: https://doi.org/10.64898/2026.06.19.733462; this version posted June 20, 2026. The copyright holder for this preprint (which was not certified by peer review) is the author/funder, who has granted bioRxiv a license to display the preprint in perpetuity. It is made available under a CC-BY 4.0 International license.

Capillary bundling of microtubules by condensates: supplementary information

Bernardo Gouveia[1†], J. Pedro de Souza[2†], Venecia Valdez[3], Joshua W. Shaevitz[4,5], Howard A. Stone[6,*], and Sabine Petry[3,*]

[1]Department of Chemical and Biological Engineering
[2]Omenn-Darling Bioengineering Institute
[3]Department of Molecular Biology
[4]Department of Physics
[5]Lewis-Sigler Institute for Integrative Genomics
[6]Department of Mechanical and Aerospace Engineering
Princeton University, Princeton, NJ 08544, USA
†These authors contributed equally.
∗To whom correspondence should be addressed: hastone@princeton.edu, spetry@princeton.edu

bioRxiv preprint doi: https://doi.org/10.64898/2026.06.19.733462; this version posted June 20, 2026. The copyright holder for this preprint (which was not certified by peer review) is the author/funder, who has granted bioRxiv a license to display the preprint in perpetuity. It is made available under a CC-BY 4.0 International license.

S1 Theoretical methods

S1.1 Capillary force between two microtubules

We first wish to estimate the dynamics at play when two microtubules adhere together under the action of a wetted condensed film with interfacial tension γ. Initially, we assume microtubules are adhered in parallel fashion, so that there is only an adhesive force that acts to drive microtubules closer together (Fig. T1a). Quite generally at low Reynolds number, the hydrodynamic drag F_d must balance the adhesive force F_a,

$$F_d = F_a. \tag{S1}$$

To estimate the adhesive force, we assume a condensed film of constant cross section between microtubules of radius R and length L spaced a distance h apart (Fig. T1a). This is a large simplification from the experimental reality, where the condensate morphology is complicated, but it should allow us to capture the basic physics. In so doing, we can use the exact result from Princen [1] to write

$$F_a = \gamma \kappa A_{\text{wet}} = -\frac{\gamma \cos(\theta + \alpha)}{h/2 - R(\cos\alpha - 1)} 2\alpha R L, \tag{S2}$$

where κ is the mean curvature, A_{wet} is the wetted area, θ is the contact angle, and α is a parameter that depends on the volume of the condensed phase in the film, and therefore is set by the bulk concentration of protein.

We consider two possible sources of drag. First is the drag microtubules must overcome to move through the solvent of viscosity μ_{s}. This is given by [2]

$$F_d = \zeta_\perp u_y \sim \frac{4\pi\mu_{\text{s}} L}{\log(L/2R)} \dot{h}, \tag{S3}$$

with perpendicular drag coefficient $\zeta_\perp$.

The second source of drag is the flow associated with squeezing the condensed film of viscosity μ as the microtubules come together. This squeezing motion mainly generates flows along the microtubule in the horizontal (x) direction perpendicular to the direction of squeezing (y). Performing a scaling analysis $x \sim b(t)$, $y \sim h(t)$, $u_x \sim U$, and $u_y \sim \dot{h}$ and demanding that continuity $\partial_x u_x + \partial_y u_y = 0$ hold at O(1) forces

$$\dot{h} \sim U\epsilon, \tag{S4}$$

where $\epsilon = h/b < 1$. We note that the assumption of $\epsilon < 1$ gets better over time as the microtubules squeeze together.

Performing the same scaling analysis on the x component of the Stokes equations $\partial_x p = \mu \nabla^2 u_x$ and neglecting terms of $\mathrm{O}(\epsilon^2)$ forces the pressure p to scale like

$$p \sim \frac{\mu U b}{h^2} \sim \frac{\mu \dot{h} b^2}{h^3}, \tag{S5}$$

bioRxiv preprint doi: https://doi.org/10.64898/2026.06.19.733462; this version posted June 20, 2026. The copyright holder for this preprint (which was not certified by peer review) is the author/funder, who has granted bioRxiv a license to display the preprint in perpetuity. It is made available under a CC-BY 4.0 International license.

where in the last step we used equation (S4). We note that this squeezing pressure is the dominant resistance to flow as $h \to 0$, since the viscous shear stress scales like $\sigma = \mu \partial_y u_x \sim \mu U/h \sim \mu \dot{h} b/h^2$.

We now need to relate b and h to each other using geometry. The profile of the microtubule as measured from the center of the liquid bridge (Fig. T1a) is $y(x) = h/2 + \sqrt{R^2 - x^2} \sim h/2 + R(1 - x^2/2R)$, where the last equality is valid if $x < R$, which is an assumption that improves as the squeeze flow progresses. Performing the scaling analysis $x \sim b$ we find that if $y \sim h$ then b must scale like

$$b \sim \sqrt{hR}. \tag{S6}$$

Putting this all together results in the drag force

$$F_d = pA_{\text{wet}} \sim \left(\frac{\mu \dot{h} b^2}{h^3}\right)(2bL) \sim \frac{2\mu R^{3/2} L}{h^{3/2}} \dot{h}. \tag{S7}$$

As the squeeze flow progresses and $h \to 0$, it is clear that equation (S7) dominates (S3). Thus, equating equation (S7) with equation (S2) results in a separable ODE for $h(t)$

$$\frac{h/2 + R(1 - \cos\alpha)}{h^{3/2}} \dot{h} = -\frac{\alpha\gamma \cos(\theta + \alpha)}{\mu\sqrt{R}}, \tag{S8}$$

which can be integrated using the initial condition $h(t = 0) = h_0$ to give an implicit equation for $h(t)$

$$\sqrt{\frac{h}{h_0}} - 1 + 2R(\cos\alpha - 1)\left(\frac{1}{\sqrt{h_0 h}} - \frac{1}{h_0}\right) = -\frac{\alpha\gamma \cos(\theta + \alpha) t}{\mu\sqrt{h_0 R}}. \tag{S9}$$

As $h(t \to \infty) \to 0$, there is a dominant balance between only two terms in equation (S9) which results in the final asymptotic scaling

$$h \sim \sqrt{\frac{\tau_f}{t}}, \tag{S10}$$

where the timescale $\tau_f = 2\mu(\cos\alpha - 1)\sqrt{R^3}/\alpha\gamma\cos(\theta + \alpha)$ emerges as the dominant capillary time that governs the squeezing dynamics. This is to say that, under action of capillary forces alone, the microtubules will get arbitrarily close to each other with a spacing between them that decreases as $\sim \sqrt{\tau_f/t}$. Of course, this theory breaks down at the length scale of single molecules, at which point a more complicated disjoining pressure arises that likely results in a finite equilibrium separation [3].

bioRxiv preprint doi: https://doi.org/10.64898/2026.06.19.733462; this version posted June 20, 2026. The copyright holder for this preprint (which was not certified by peer review) is the author/funder, who has granted bioRxiv a license to display the preprint in perpetuity. It is made available under a CC-BY 4.0 International license.

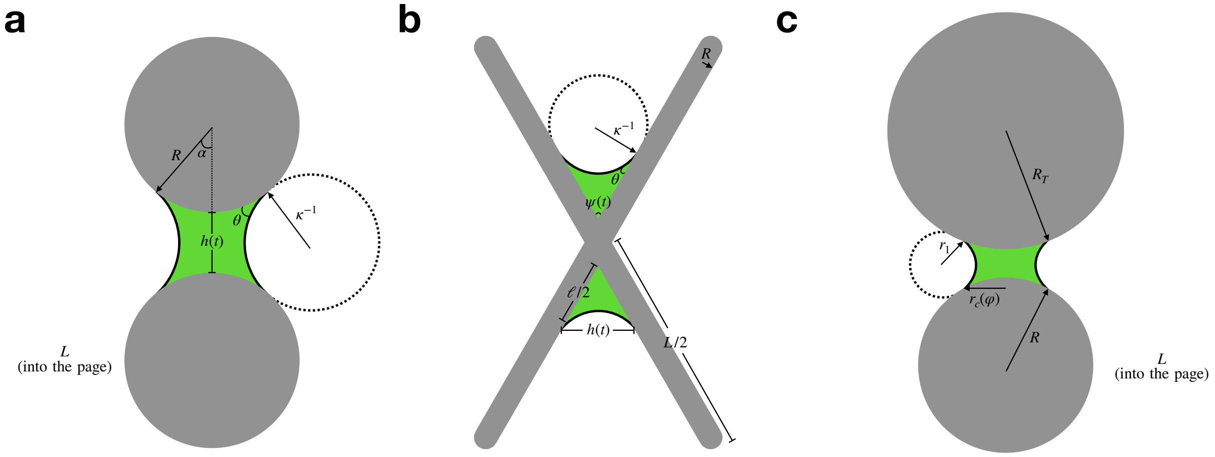

Figure T1: (a) Model for capillary force between two microtubules. Two parallel microtubules of length L and radii R are adhered together by a capillary bridge of constant cross-sectional area along the length of the microtubule. (b) Model for capillary torque between two microtubules. Two microtubules meet at an acute angle $\psi(t)$ hinged together by a capillary bridge of wetted length ℓ. (c) Model for capillary force between a spherical AFM tip of radius R_T and cylindrical microtubule of radius R assuming perfect wetting ($\theta = 0$).

S1.2 Capillary torque between two microtubules

We now move to consider the rotational dynamics at play when two microtubules contact each other at an acute angle ψ (Fig. T1b). At low Reynolds number, the adhesive capillary torque T_a must balance the resistive viscous torque T_d,

$$T_d = T_a. \tag{S11}$$

An exact calculation of T_a is complex and depends on the intricate details of the highly contorted interface between angled filaments [4, 5]. However, to make analytical progress we argue that the capillary pressure that drives filaments to snap together is dominated by the in-plane radius of curvature (Fig. T1b). The out-of-plane radius of curvature serves principally to decrease the out-of-plane distance h between filaments, which was already considered in Sec. (S1.1) (Fig. T1a). Because $L \gg h$, it is reasonable to carry out the calculation entirely in the 2d plane. Using plane geometry (Fig. T1b), the radius of curvature is therefore $\kappa^{-1} = -\ell \sin(\psi/2)/2\cos(\psi/2 + \theta)$, where ℓ is the wetted length of the droplet on the filaments that drives the snapping dynamics. This gives rise to the capillary force

$$F_a = \gamma \kappa A_{\text{wet}} \sim -\frac{2\gamma \cos(\psi/2 + \theta)}{\ell \sin(\psi/2)} 2\pi R \ell = -\frac{4\pi\gamma R \cos(\psi/2 + \theta)}{\sin(\psi/2)}, \tag{S12}$$

bioRxiv preprint doi: https://doi.org/10.64898/2026.06.19.733462; this version posted June 20, 2026. The copyright holder for this preprint (which was not certified by peer review) is the author/funder, who has granted bioRxiv a license to display the preprint in perpetuity. It is made available under a CC-BY 4.0 International license.

where for simplicity we assume the condensate wets the entire circumference of the microtubule. The corresponding torque is

$$T_a = \int_0^{\ell/2} \mathrm{d}l \; F_a \sim -\frac{2\pi\gamma R\ell \cos(\psi/2+\theta)}{\sin(\psi/2)}. \tag{S13}$$

For the viscous torque, we must consider two dynamical regimes. The first regime is where $\psi(t)$ is close to the initial angle ψ_0, which can be large and finite as observed in our experiments. In this case, the wetted length ℓ satisfies $\ell \ll L$ and can be treated as a constant $\ell = \ell_0$ throughout the motion. The resistance to snapping is dominated by rotating microtubules of length L through the solvent of viscosity μ_s (Fig. T1b), which is given by [2]

$$T_d = \zeta_r \Omega \sim \frac{\pi \mu_\mathrm{s} L^3}{3\log(L/2R)}\dot{\psi}. \tag{S14}$$

Equating equations (S13) and (S14) results in the ODE for $\psi(t)$

$$\tau_1 \dot{\psi} = -\frac{\cos(\psi/2+\theta)}{\sin(\psi/2)}, \tag{S15}$$

where the timescale that governs the early time snapping dynamics falls out as $\tau_1 = \frac{\mu_\mathrm{s} L^3}{6\gamma R \ell_0 \log(L/2R)}$. We can integrate this ODE to give an implicit equation for $\psi(t)$. Taking $\psi(t=0) = \psi_0$, we find

$$\frac{t}{\tau_1} = \frac{1}{2}(\psi - \psi_0)\sin\theta + \log\left[\frac{\cos(\psi/2+\theta)}{\cos(\psi_0/2+\theta)}\right]\cos\theta. \tag{S16}$$

If we assuming perfect wetting $\theta = 0$, a reasonable assumption for condensed MAPs on microtubules, we find the following explicit solution for $\psi(t)$,

$$\psi(t) = 2\arccos\left[\cos(\psi_0/2)e^{t/\tau_1}\right], \tag{S17}$$

which can be expanded for early times $t \to 0$ dropping terms of $\mathrm{O}(t^2)$ and higher to find

$$\psi(t) \sim \psi_0 - \frac{2}{\tan(\psi_0/2)\tau_1}t. \tag{S18}$$

Even though equation (S18) was derived in the $\psi(t \to 0) \to \psi_0$ limit, this linear function can represent most of the experimentally measured data in Fig. 2c well, and predicts that microtubules will take a time $T_1 = (\psi_0/2)\tan(\psi_0/2)\tau_1$ to fully snap together.

The second regime is where $\psi(t) \to 0$, when the microtubules are snapping together and have to squeeze the condensate of viscosity μ between the aligning microtubules (Fig. T1b). In this case, $\ell = \ell(t)$ as the wetted length moves significantly along the microtubules during the squeeze flow. Taking $h(t)$ as the horizontal distance between

bioRxiv preprint doi: https://doi.org/10.64898/2026.06.19.733462; this version posted June 20, 2026. The copyright holder for this preprint (which was not certified by peer review) is the author/funder, who has granted bioRxiv a license to display the preprint in perpetuity. It is made available under a CC-BY 4.0 International license.

snapping filaments (Fig. T1b), the same arguments that resulted in equation (S5) can be applied here, such that the dominant viscous resistance is the squeezing pressure which scales like

$$p \sim \frac{\mu \dot{h} \ell^2}{h^3}, \tag{S19}$$

so long as $h < \ell$, an assumption that improves as the squeeze flow progresses as $\psi \to 0$. To make progress, we relate h to ψ and ℓ using the law of cosines

$$h^2 = \frac{\ell^2}{2}(1-\cos\psi) \implies h \sim \frac{\ell}{\sqrt{2}}\sqrt{1-(1-\psi^2/2)} = \frac{\ell\psi}{2}, \tag{S20}$$

where the second relation is valid for small ψ. Therefore $\dot{h} = (\dot{\ell}\psi + \ell\dot{\psi})/2$. We can further relate ℓ and ψ by employing the constraint that the condensate volume remains constant during the motion. In this 2D calculation, we can approximately represent this by the triangular area A enclosed by h and the two wetted lengths $\ell/2$. Computing the area of the condensate exactly would result in an unwieldy elliptic integral that is not needed to model the dominant physics of h decreasing as ℓ increases during the snapping dynamics. The simple triangular geometry therefore results in $A = (\ell^2/8)\sin\psi \sim (\ell^2/8)\psi$ for small ψ. Therefore

$$\dot{A} = 0 \implies \frac{\dot{\ell}}{\ell} = -\frac{\dot{\psi}}{2\psi}. \tag{S21}$$

Substituting this into the expression for $\dot{h}$ gives $\dot{h} = (\dot{\ell}\psi + \ell\dot{\psi})/2 = \left(1 - \frac{1}{2\psi}\right)\frac{\ell\dot{\psi}}{2} \sim -\frac{\ell\dot{\psi}}{4\psi}$ for small ψ. Substituting these results into equation gives

$$p \sim -\frac{2\mu\dot{\psi}}{\psi^4}, \tag{S22}$$

and therefore the resulting force is

$$F_d = pA_{\text{wet}} \sim -\frac{2\mu\dot{\psi}}{\psi^4}(2\pi R\ell), \tag{S23}$$

from which the viscous torque may be computed as

$$T_d = \int_0^{\ell/2} \mathrm{d}l \; F_d \sim -\frac{\pi\mu R\ell^2\dot{\psi}}{2\psi^4}. \tag{S24}$$

We can expand the capillary torque in equation (S13) for $\psi \to 0$ to give

$$T_a \sim -2\pi\gamma R\ell\left(\frac{\cos\theta - \sin\theta(\psi/2)}{\psi/2}\right) \sim -\frac{4\pi\gamma R\ell\cos\theta}{\psi}. \tag{S25}$$

bioRxiv preprint doi: https://doi.org/10.64898/2026.06.19.733462; this version posted June 20, 2026. The copyright holder for this preprint (which was not certified by peer review) is the author/funder, who has granted bioRxiv a license to display the preprint in perpetuity. It is made available under a CC-BY 4.0 International license.

Equating equations (S24) and (S25) gives us our final ODE for $\psi(t)$

$$\frac{\ell\dot{\psi}}{\psi^3} = \frac{8\gamma\cos\theta}{\mu}. \tag{S26}$$

At this stage it is most convenient to eliminate ℓ in favor of ψ by integrating the geometric constraint equation (S21). Taking $\psi(t=0)=\psi_0$ and $\ell(t=0)=\ell_0$, we have

$$\begin{aligned}
\frac{\dot{\ell}}{\ell} + \frac{\dot{\psi}}{2\psi} &= 0 && \text{(S27a)}\\
\frac{\mathrm{d}}{\mathrm{d}t}\left(\log\ell + \log\sqrt{\psi}\right) &= 0 && \text{(S27b)}\\
\log\left(\frac{\ell}{\ell_0}\right) &= \log\left(\frac{\sqrt{\psi_0}}{\sqrt{\psi}}\right) && \text{(S27c)}\\
\ell &= \ell_0\sqrt{\frac{\psi_0}{\psi}}. && \text{(S27d)}
\end{aligned}$$

Substituting this result into equation (S26) gives

$$\frac{\dot{\psi}}{\psi^{7/2}} = \frac{8\gamma\cos\theta}{\ell_0\sqrt{\psi_0}\mu}, \tag{S28}$$

which can be directly integrated to give

$$\frac{1}{\psi_0^{5/2}} - \frac{1}{\psi^{5/2}} = \frac{t}{\tau_2}, \tag{S29}$$

where $\tau_2 = \frac{\mu\ell_0\sqrt{\psi_0}}{20\gamma\cos\theta}$ is the timescale that governs the late time snapping dynamics. As $\psi(t\to\infty)\to 0$ a dominant balances emerges resulting in the final asymptotic scaling

$$\psi \sim \left(\frac{\tau_2}{t}\right)^{2/5}. \tag{S30}$$

While eventually we expect this lubrication scaling to dominate for late times, we do not have the experimental spatiotemporal resolution to observe the relavant small angle dynamics in this work.

S1.3 Comparing mesoscale capillary bridges to single-molecule crosslinkers

To more solidly contrast snapping mechanisms of crosslinking and capillary forces, the dynamics based on crosslinking can be analyzed more precisely. If the crosslinkers act as

bioRxiv preprint doi: https://doi.org/10.64898/2026.06.19.733462; this version posted June 20, 2026. The copyright holder for this preprint (which was not certified by peer review) is the author/funder, who has granted bioRxiv a license to display the preprint in perpetuity. It is made available under a CC-BY 4.0 International license.

effective springs that connect when the distance between opposing microtubules is less than $2b$ and supply a spring force per cross linker of $f_{cl} = -kx$, where x is the distance between neighboring microtubule surfaces, $x = l\sin(\psi/2)$, with l describing a coordinate parallel with the microtubule, and k a spring constant. With a crosslinking density per unit length, q, the force in the x direction would be

$$f_{cl} = -qkl\sin(\psi/2) \quad \text{(S31)}$$

The adhesive torque would then be:

$$T_{cl} = \int_0^{b/\sin(\psi/2)} \mathrm{d}l \; l f_{cl}\cos(\psi/2) = -\frac{qkb^3}{3}\frac{\cos(\psi/2)}{\sin^2(\psi/2)}. \quad \text{(S32)}$$

Here, $b/\sin(\psi/2)$ describes the distance over which the two crossed microtubules will be connected by crosslinkers.

Again, we can balance the adhesive torque with the torque required to rotate a microtubule of length L, T_d. Since here there is no viscous condensate driving the adhesion, we assume that the dominant viscous torque is the one needed to rotate the microtubule in the solvent of viscosity μ_s,

$$T_d = \zeta_r\Omega \sim \frac{\pi\mu_{\mathrm{s}}L^3}{3\log(L/2R)}\dot{\psi}, \quad \text{(S33)}$$

to arrive at the differential equation governing snapping:

$$\tau_3\dot{\psi} = -\frac{\cos(\psi/2)}{\sin^2(\psi/2)} \quad \text{(S34)}$$

where $\tau_3 = \frac{\pi\mu_{\mathrm{s}}L^3}{qkb^3\log(L/2R)}$.

We are interested in the dynamics of $\psi(t)$ between $\psi(t=0) = \psi_0$ and when the entirety of the microtbule is connected, $\psi = 2\sin^{-1}(2R_0/L)$. Here, we will assume that $R_0 \ll L$.

The solution to the differential equation gives an implicit expression for $\psi(t)$

$$\frac{t}{\tau_3} = -2\tanh^{-1}\left(\sin\left(\frac{\psi}{2}\right)\right) + 2\sin\left(\frac{\psi}{2}\right) + 2\tanh^{-1}\left(\sin\left(\frac{\psi_0}{2}\right)\right) - 2\sin\left(\frac{\psi_0}{2}\right), \quad \text{(S35)}$$

which if ψ is always is small, can be simplified to:

$$\psi = \left(-\frac{12t}{\tau_3} + \psi_0^3\right)^{1/3} \quad \text{(S36)}$$

This time course function shape appears quite similar to the time course predicted for the first regime of capillary snapping. From the shape of the snapping curves, it is difficult

bioRxiv preprint doi: https://doi.org/10.64898/2026.06.19.733462; this version posted June 20, 2026. The copyright holder for this preprint (which was not certified by peer review) is the author/funder, who has granted bioRxiv a license to display the preprint in perpetuity. It is made available under a CC-BY 4.0 International license.

to differentiate directly between mechanisms of capillary snapping driven on a time scale τ_1 and τ_3.

We can instead compare the different expectations for the timescales of snapping based on ratios of these effective time scales, for example:

$$\frac{\tau_1}{\tau_3} = \frac{qkb^3}{6\pi\gamma R\ell}, \tag{S37}$$

where we have now substituted $\ell_0 \to \ell$. If we assume similar adhesion force scales between the two models so $kb \approx \gamma R$, and we take $qb \approx 1$, we find that the ratio of time scales is sensitively governed by the ratio b/ℓ. b is always a molecular scale, whereas ℓ is at the scale of the wetting film, which can reach the order of the microtubule length L for late times, $b \ll \ell$. Because b is a molecular length scale and l is the mesoscopic length scale of the wetted film, we expect $\tau_1 \ll \tau_3$. That is, even for comparable adhesion forces, we generally expect capillary torques to be at least an order of magnitude larger than crosslinking torques due to their mesoscale nature. This means that the crosslinking dynamics are expected to act much more slowly than the capillary snapping, although the system-specific details will determine by how much.

S1.4 Capillary force between a spherical tip and a microtubule

Here, we estimate the force between a sphere (to represent the AFM tip) and a cylinder (to represent the microtubule) with a connecting capillary bridge within the Derjaguin approximation [6] and assuming perfect wetting. We assume that the sphere of radius R_T is in contact with a cylinder with radius R, which we define as the origin of our coordinate system. A condensate bridge with constant radius of curvature r_1 perfectly wets the two surfaces. The Laplace pressure will be approximately:

$$\Delta P = -\frac{\gamma}{r_1}, \tag{S38}$$

as long as the contact area is small relative to the radii of the confining surfaces and the contact line at $r_c(\varphi)$ is much larger than r_1. At equilibrium, the radius of curvature and contact angles will be fixed regardless of the azimuthal angle from the origin.

We can define the bottom surface of a sphere with a function,

$$z = R_T - \sqrt{R_T^2 - r^2}, \tag{S39}$$

and similarly for the cylinder

$$z = -R + \sqrt{R^2 - r^2\cos^2\varphi}, \tag{S40}$$

where r and φ are given by cylindrical coordinates centered at the origin. If we assume that the capillary bridge is small relative to R_T and R at leading order, $r \ll R_T, R$, then

bioRxiv preprint doi: https://doi.org/10.64898/2026.06.19.733462; this version posted June 20, 2026. The copyright holder for this preprint (which was not certified by peer review) is the author/funder, who has granted bioRxiv a license to display the preprint in perpetuity. It is made available under a CC-BY 4.0 International license.

the distance between the two surfaces can be approximated as

$$h = \frac{r^2}{2R_T} + \frac{r^2 \cos^2 \varphi}{2R}. \tag{S41}$$

Based on the perfect wetting assumption, we have $h = 2r_1$. Suppose that the contact line exists at some angular-dependent position $r_c(\varphi)$. In order for the system to be at equilibrium, the value of r_1 must be fixed at every angle. From this requirement, we get a definition for $r_c(\varphi)$

$$r_c^2(\varphi) = \frac{4r_1}{\frac{1}{R_T} + \frac{\cos^2(\varphi)}{R}}. \tag{S42}$$

In order to find the capillary force, we need to compute the product of the Laplace pressure multiplied by the area of contact. The area of contact, A, can be calculated by direct integration,

$$A = \frac{1}{2}\int_0^{2\pi} r_c^2(\varphi) d\varphi = \frac{4\pi r_1 \sqrt{R_T}}{\sqrt{\frac{1}{R_T} + \frac{1}{R}}}. \tag{S43}$$

Therefore, the capillary force at contact, F, can be approximated as:

$$F = -4\pi\gamma\bar{R}, \qquad \bar{R} = \frac{\sqrt{R_T}}{\sqrt{\frac{1}{R_T} + \frac{1}{R}}}. \tag{S44}$$

In the limit of $R \to \infty$ the expression for a sphere interacting with a plane is recovered. In the limit of $R_T \to \infty$, the force diverges since the capillary bridge extends over the entire (infinite) cylinder.

S1.5 Phase field model of the condensate interface

S1.5.1 Flory-Huggins Cahn-Hilliard Model

Here, we employ the standard mean-field Flory-Huggins [7, 8] model to represent the protein (biopolymer) thermodynamics, and we combine the homogeneous part with a Cahn-Hilliard [9] term to capture the surface energy. The dimensionlesss free energy density $\tilde{f}$, at temperature T, monomer and solvent volume v, and with Boltzmann constant k_B is

$$\begin{aligned}\tilde{f} = \frac{fv}{k_BT} =& \frac{1}{N}\phi\ln(\phi) + (1-\phi)\ln(1-\phi) \\ &+ \chi\phi(1-\phi) + \frac{\ell^2}{2}|\nabla\phi|^2,\end{aligned} \tag{S45}$$

where ϕ is the volume fraction of the protein, N is its length, χ is the Flory interaction parameter, and ℓ is a length scale that characterizes the phase boundary thickness that

bioRxiv preprint doi: https://doi.org/10.64898/2026.06.19.733462; this version posted June 20, 2026. The copyright holder for this preprint (which was not certified by peer review) is the author/funder, who has granted bioRxiv a license to display the preprint in perpetuity. It is made available under a CC-BY 4.0 International license.

is on the scale of an individual monomer. From this point forward, the gradients of composition will be assumed to only occur in one coordinate direction, x, such that $|\nabla\phi|^2 = |\phi'|^2$.

In order to derive an analytical expression for the surface energy, we will expand the free energy density around its critical point and truncate at a few terms. The critical point, where $f'''(\phi) = 0$, is defined at a composition of ϕ_c and interaction strength χ_c,

$$\phi_c = \frac{1}{1+\sqrt{N}}, \quad \chi_c = \frac{1}{2}\left(1 + \frac{1}{\sqrt{N}}\right)^2. \tag{S46}$$

The free energy density expanded around the critical point up to fourth order in $\delta\phi = \phi - \phi_c$ and neglecting terms that are constant or linear in ϕ gives

$$\tilde{f} \approx -(\chi - \chi_c)\delta\phi^2 + \frac{\chi_c^2\sqrt{N}}{3}\delta\phi^4 + \frac{\ell^2}{2}\delta\phi'^2. \tag{S47}$$

We can derive a generalized chemical potential that includes contributions from the interface by taking a variational derivative, $\mu = \delta\tilde{f}/\delta(\delta\phi)$,

$$\mu \approx -2(\chi - \chi_c)\delta\phi + \frac{4\chi_c^2\sqrt{N}}{3}\delta\phi^3 - \ell^2\delta\phi''. \tag{S48}$$

Near the critical point, the homogeneous part of the chemical potential is an odd function. Chemical equilibrium between the dilute and concentrated phase implies that: $\delta\phi_+ = -\delta\phi_-$. Therefore, we have in the bulk phases near the critical point [10],

$$\delta\phi_\pm = \pm\sqrt{\frac{3}{2}}\frac{(\chi - \chi_c)^{1/2}}{N^{1/4}\chi_c}, \quad \mu = 0. \tag{S49}$$

Next, we solve for the 1D profile across the phase boundary between composition $\delta\phi_-$ as $x \to -\infty$ and composition $\delta\phi_+$ as $x \to +\infty$, relative to the interface at $x = 0$, by applying $\mu = 0$ at all values of x. We define the following rescalings into dimensionless variables $\delta\tilde{\phi}$ and $\tilde{x}$ to simplify the calculations,

$$\delta\tilde{\phi} = \frac{\delta\phi}{\left(\sqrt{\frac{3}{2}}\frac{(\chi-\chi_c)^{1/2}}{N^{1/4}\chi_c}\right)}, \quad \tilde{x} = \frac{x}{\ell}(\chi - \chi_c)^{1/2} \tag{S50}$$

to render the equilibrium chemical potential equation as:

$$\frac{d^2\delta\tilde{\phi}}{dx^2} = -2\delta\tilde{\phi}(1 - \delta\tilde{\phi}^2), \quad \delta\tilde{\phi}(\pm\infty) = \pm 1. \tag{S51}$$

The solution to the above equation is:

$$\delta\tilde{\phi} = \tanh(\tilde{x}), \tag{S52}$$

bioRxiv preprint doi: https://doi.org/10.64898/2026.06.19.733462; this version posted June 20, 2026. The copyright holder for this preprint (which was not certified by peer review) is the author/funder, who has granted bioRxiv a license to display the preprint in perpetuity. It is made available under a CC-BY 4.0 International license.

or with dimensions now returned

$$\phi = \frac{1}{1+\sqrt{N}} + \sqrt{\frac{3}{2}}\frac{(\chi-\chi_c)^{1/2}}{N^{1/4}\chi_c}\tanh\left(\frac{x}{\ell}(\chi-\chi_c)^{1/2}\right). \tag{S53}$$

The surface tension can be derived by integrating the excess free energy over the interface region [9],

$$\gamma = \frac{k_B T}{v}\int_{-\infty}^{\infty}\left(\tilde{f}(x) - \tilde{f}(\infty)\right)dx, \tag{S54}$$

or in in other terms

$$\gamma = \frac{3k_B T\ell(\chi-\chi_c)^{3/2}}{2vN^{1/2}\chi_c^2}\int_{-\infty}^{\infty}\left(\frac{1}{2} - \delta\tilde{\phi}^2(x) + \frac{1}{2}\delta\tilde{\phi}^4 + \frac{1}{2}\delta\tilde{\phi}'^2\right)d\tilde{x}. \tag{S55}$$

We can evaluate the integral analytically to get a final expression for the surface tension in the mean-field limit,

$$\gamma = \frac{2k_B T\ell(\chi-\chi_c)^{3/2}}{vN^{1/2}\chi_c^2}. \tag{S56}$$

The above expression highlights that the surface tension increases with increasing interaction strength, χ, but decreases as the protein sequence length, N, increases. Next, we rationalize the possible trends in χ in the context of electrostatic interactions, similar to ref. [11], since charge interactions can strongly influence phase separation and thus surface tension of condensates.

S1.5.2 Estimating the electrostatic contribution to the free energy density

In what follows, we will derive the leading-order contribution of electrostatic interactions to the Flory interaction parameter, χ, and thereby connect the protein charge interactions to the surface tension of their condensed phases. The Voorn-Overbeek [12, 13] model combines the Flory-Huggins model of polymer entropy with a Debye-Huckel type free energy density from the screening by ions in an electrolyte. The electrostatic contribution to the free energy density is given by

$$\tilde{f}_{\text{el}} = \frac{vf_{\text{el}}}{k_B T} = -\frac{v\kappa_D^3}{12\pi}, \tag{S57}$$

where κ_D is the inverse Debye length. The protein is treated as an unconnected polyelectrolyte, such that it also contributes to the ionic strength of the solution. Therefore, the inverse Debye length is defined as,

$$\kappa_D = \sqrt{4\pi\ell_B\sum_i z_i^2\theta_i\phi_i/v}, \tag{S58}$$

bioRxiv preprint doi: https://doi.org/10.64898/2026.06.19.733462; this version posted June 20, 2026. The copyright holder for this preprint (which was not certified by peer review) is the author/funder, who has granted bioRxiv a license to display the preprint in perpetuity. It is made available under a CC-BY 4.0 International license.

where the summation over species i corresponds to all species, including the salt ions and protein. ℓ_B is the Bjerrum length which describes the length at which two point charges interact with energy k_BT, defined as

$$\ell_B = \frac{e^2}{4\pi\epsilon_r\epsilon_0 k_B T} \tag{S59}$$

where e is an elementary charge, ϵ_r is the dielectric permittivity of the solvent, and ϵ_0 is the permittivity of free space.

Here, we are assuming that all monomers and salt ions occupy the same volume, v. θ_i corresponds to the total fraction of charged sites on each species (the sum of both the number of positive and negative sites), where we assume the salt ions are completely dissociated $\theta_\pm = 1$. Further, we assume that all charged sites on the protein carry a charge of $z_i \pm 1$.

While the model is able to capture basic trends in the salt-dependent phase separation of polyelectrolytes and their electrostatic interactions, it is not a perfect model for concentrated electrolytes. Further, it neglects the correlations in the charge sequences along the protein [14] and structuring in the electrolyte [13]. Therefore, we use this model to provide general relationships with respect to protein charge, but we do not expect it to be quantitatively accurate for all model systems.

The free energy density can be recast in the convenient form,

$$\tilde{f}_{\mathrm{el}} = -\alpha \left(\sum_i \theta_i \phi_i \right)^{3/2}, \tag{S60}$$

where the parameter α is defined by

$$\alpha = \frac{2}{3}\sqrt{\frac{\pi \ell_B^3}{v}} \tag{S61}$$

To find the leading order electrostatic contributions to the free energy density, we can expand the result in the limit of small protein volume fraction. If we expand the free energy density around a protein concentration of $\phi_p = 0$, we get the Flory-Huggins model with a salt dependent χ contribution, χ_{el}.

$$\tilde{f}_{\mathrm{el}} \approx -\chi_{\mathrm{el}} \phi_p^2 \tag{S62}$$

where:

$$\chi_{\mathrm{el}} = \frac{3\alpha\theta_p^2}{8\sqrt{\sum_s z_i^2 \phi_i}}. \tag{S63}$$

The summation in the denominator is now only over the salt ion species. Here, we see that the leading order electrostatic contribution to the Flory interaction parameter goes

bioRxiv preprint doi: https://doi.org/10.64898/2026.06.19.733462; this version posted June 20, 2026. The copyright holder for this preprint (which was not certified by peer review) is the author/funder, who has granted bioRxiv a license to display the preprint in perpetuity. It is made available under a CC-BY 4.0 International license.

with the square of protein charge and decreases inversely proportional to the square root of salt concentration. Note that the actual value of χ may include some non-electrostatic contribution, χ_0, so that

$$\chi = \chi_0 + \chi_{\mathrm{el}}. \tag{S64}$$

To compare BuGZ and TPX2, the total fraction of charged residues, θ_p, were estimated from the amino acid sequences using the EMBOSS pK values [15, 16] for the charged residues at pH 6.8 of the BRB80 buffer. For TPX2, $\theta_p = 0.33$ and for BuGZ, $\theta_p = 0.20$. [1]

In the physiological solutions at room temperature, $\ell_B = 0.7$ nm, and we can roughly assume that $\ell = 0.3$ nm and $v = (0.3\mathrm{nm})^3$ to characterize the size of solvent, monomers, and salt ions. With these parameters, the value of α is 4.2. The BRB80 electrolyte has an ionic strength of about 160 mM. Therefore, the values of χ_{el} for TPX2 and BuGZ, respectively, are 2.3 and 0.9. Note that these predicted values are quite sensitive to the chosen parameters ℓ and v, although their ratio will not change significantly.

To estimate the expected difference in surface tension between TPX2 and BuGZ, we only need to define N based on the sequence length. The values of N for TPX2 and BuGZ are 694 and 959 respectively. The mean-field theory therefore would predict that from electrostatic interactions alone, the surface tension of TPX2 and BuGZ should roughly differ by a factor of 10, which is what we observed experimentally. Even so, the mean-field theory overpredicts the absolute value of γ by two orders of magnitude, highlighting the possible inaccuracies introduced through the expansions and truncations around the critical point.

S1.6 Sliding resistance from a viscous film

Here we consider the hydrodynamic resistance experienced by parallel microtubules sliding apart due to a constant force F. In our experiments, this force is provided by Eg5 motors sliding one mobile microtubule relative to another that is chemically fixed to the surface.

In the case where there is just solvent of viscosity μ_s between the microtubules, the resistance to sliding is dominated by the classic slender-body result $F_s = \frac{2\pi\mu_s L}{\log(L/2R)} V_s$, where V_s is the measured sliding speed in this case.

In the case where there is an additional condensed film of viscosity μ between the microtubules, the motion is resisted by both the slender-body term as well as the viscous film of thickness h. The simplest approximation for this additional resistance that captures the basic features is $\frac{\mu V_f}{h} A_{\mathrm{wet}} \approx \frac{\mu V_f}{h} \pi R L$, and therefore $F_f = \left[\frac{2\pi\mu_s L}{\log(L/2R)} + \frac{\mu\pi R L}{h}\right] V_f$, where V_f is the measured sliding speed in this case.

Assuming the Eg5 driving force is the same in both scenarios, we can set $F_s = F_f$ and compute the ratio V_f/V_s, which serves as a prediction for how much the condensed film

[1] In the main text, the symbol f is used in place of θ_p to avoid confusion with the contact angle, θ.

bioRxiv preprint doi: https://doi.org/10.64898/2026.06.19.733462; this version posted June 20, 2026. The copyright holder for this preprint (which was not certified by peer review) is the author/funder, who has granted bioRxiv a license to display the preprint in perpetuity. It is made available under a CC-BY 4.0 International license.

slows down the Eg5-driven sliding,

$$\frac{V_f}{V_s} = \frac{1}{1 + \frac{\mu R \log(L/2R)}{2\mu_s h}}. \tag{S65}$$

While these are rather simple scaling arguments, we believe they capture the basic physics. The real drag expression in the presence of the condensed film results from a complicated multiphase flow problem, and since $R < h$ simplifying lubrication approximations do not apply. Such an effort would be outside the scope of this work

S2 Experimental methods

S2.1 Protein expression and purification

Full-length TPX2 (*X. laevis*, Gene ID: 398174) with a N-terminal StrepII-6xHis-GFP-TEV tag was cloned into a pST50 vector, transformed into Rosetta2 *E. Coli* cells, grown up to an $OD_{600} \approx 0.6$ at 37°C in 2 L of LB broth, and then expressed using 0.75 mM IPTG at 25°C for 7 hr. Cells were pelleted, homogenized, then lysed using an Emulsiflex in lysis buffer (50 mM Tris-HCl, 750 mM NaCl, 15 mM imidazole, 6 mM BME, pH 8.0) containing 2.5 mM PMSF, 10 μg/ml DNAse I, and 2 cOmplete EDTA-free protease inhibitor tablet. Lysate was centrifuged at 30,000 RPM on a 45 Ti rotor for 30 min and the supernatant was bound to Ni-NTA agarose beads equilibrated in lysis buffer. Protein was eluted with lysis buffer containing 200 mM imidazole and then further purified using a Superdex 200 HiLoad 16/600 SEC column into storage buffer (10 mM HEPES, 500 mM KCl, 1 mM MgCl2, 5 mM EGTA, 10% w/v sucrose, pH 7.7).

Full-length BuGZ (*H. sapiens*, Gene ID: 7756) with a C-terminal StrepII-6xHis-BFP-TEV tag was cloned into a pST50 vector, transformed into Rosetta2 *E. Coli* cells, grown up to an $OD_{600} \approx 0.6$ at 37°C in 2 L of LB broth, and then expressed using 0.60 mM IPTG at 16°C for 16 hr. Cells were pelleted, homogenized, then lysed using an Emulsiflex in lysis buffer (50 mM Tris-HCl, 500 mM KCl, 1 mM MgCl2, 1 mM DTT, pH 7.8) containing 200 μM PMSF, 10 μg/ml DNAse I, and 2 cOmplete EDTA-free protease inhibitor tablet. Lysate was centrifuged at 30,000 RPM on a 45 Ti rotor for 30 min and the supernatant was bound to CV = 5 mL of equilibrated Strep-Tactin resin in lysis buffer. After a 1 hr incubation on a shaker, the mixture was transferred to a column and washed with 10 CV of lysis buffer. Protein was eluted with 3 CV of lysis buffer containing 2.5 mM desthiobiotin and then further purified using a Superdex 75 10/300 SEC column into storage buffer (25 mM HEPES, 500 mM KCl, 1 mM MgCl2, 1 mM DTT, 15% v/v glycerol, pH 7.7).

Full-length kinesin-5 (Eg5) ((*X. laevis*, Gene ID: 379112)) with a C-terminal StrepII-6xHis-GFP-TEV tag was cloned into a pFastBac vector which was used to infect 1 L of *S. frugiperda* (Sf9) cells for a 2 day expression. Virus-infected cells were pelleted then lysed using an Emulsiflex in lysis buffer (50 mM HEPES, 300 mM KCl, 1 mM MgCl2, 1 mM

bioRxiv preprint doi: https://doi.org/10.64898/2026.06.19.733462; this version posted June 20, 2026. The copyright holder for this preprint (which was not certified by peer review) is the author/funder, who has granted bioRxiv a license to display the preprint in perpetuity. It is made available under a CC-BY 4.0 International license.

DTT, 0.2 mM Mg-ATP, pH 8.0) containing 200 μM PMSF, 10 mug/ml DNAse I, and 1 cOmplete EDTA-free protease inhibitor tablet. Lysate was centrifuged at 50,000 RPM on a 70 Ti rotor for 50 min and the supernatant was bound to CV = 2 mL of equilibrated Strep-Tactin resin in lysis buffer. After a 1 hr incubation on a shaker, the mixture was transferred to a column and washed with 10 CV of lysis buffer. Protein was eluted with 3 CV of lysis buffer containing 2.5 mM desthiobiotin and then further purified using a Superose 6 10/300 SEC column into storage buffer (25 mM HEPES, 500 mM KCl, 1 mM MgCl2, 1 mM DTT, 15% v/v glycerol, pH 7.7).

All purified recombinant proteins were flash frozen in working aliquots using liquid nitrogen and stored at −80°C.

S2.2 Stabilized microtubule seed preparation

To make short, double-cycled, GMPCPP-stabilized, Atto-647 labelled microtubule seeds, a 40 μL mixture of 20 μM bovine tubulin (10% labelled with Atto-647 dye) and 1 mM GMPCPP in BRB80 (80 mM PIPES, 1 mM MgCl2, 1 mM EGTA, pH 6.8) was polymerized for 45 min in a 37°C water bath and centrifuged at 126000 g for 8 min at 30°C in a TLA-100 rotor. The supernatant was discarded and the seeds were resuspended in cold BRB80 and allowed to depolymerize on ice for 20 min. GMPCPP was then added up to 1 mM and the mixture was polymerized again for 45 min in a 37°C water bath and centrifuged at 126000 g for 8 min at 30°C in a TLA-100 rotor. The supernatant was discarded and the seeds were resuspended in room temperature BRB80. The seeds were pipetted into 1 μL aliquots, flash frozen in liquid nitrogen, and stored at −80°C.

To make long, GMPCPP-stabilized, biotinylated, Alexa-568 labelled microtubule seeds, a 40 μL mixture of 20 μM bovine tubulin (10% labelled with Alexa-568 dye, 10% labelled with biotin) and 1 mM GMPCPP in BRB80 was polymerized for 2 hr in a 37°C water bath and centrifuged at 13000 RPM for 8 min at room temperature in a tabletop centrifuge. The supernatant was discarded and the seeds were resuspended with room temperature BRB80 containing 1 mM GMPCPP. The seeds were incubated overnight in the dark at room temperature and were used within 2-3 days.

S2.3 *In vitro* bulk condensation assay

Purified protein (either GFP-TPX2 or BuGZ-BFP) was precleared of aggregates by centrifugation at 80000 RPM for 10 min at 4°C in a TLA-100 rotor. The supernatant was diluted to a target concentration in assay buffer (25 mM HEPES, 25 mM KCl (low salt) or 100 mM KCl (high salt), 1 mM MgCl2, 50 μg/ml κ-casein, pH 7.7), mixed thoroughly, and pipetted into a flow chamber. The flow chamber was sealed with nail polish and incubated coverslip side down for 10 minutes to allow condensates to settle. Condensates were imaged using epifluorescence on a Nikon Ti-E microscope with a 100x objective and 1.49 numerical aperture. Exposure times and LED power were consistent across all tested

bioRxiv preprint doi: https://doi.org/10.64898/2026.06.19.733462; this version posted June 20, 2026. The copyright holder for this preprint (which was not certified by peer review) is the author/funder, who has granted bioRxiv a license to display the preprint in perpetuity. It is made available under a CC-BY 4.0 International license.

concentrations for each protein. An ORCA-Fusion BT digital CMOS camera was used for acquisition.

Phase diagrams were computed as the area fraction of the condensed phase versus bulk concentration. The condensed phase was defined as regions having an intensity value above the threshold calculated using Otsu's method [17] for the highest concentration images for each protein.

S2.4 *In vitro* bundling assay

Double-cycled, Atto-647 microtubule seeds were diluted in assay buffer (25 mM HEPES, 100 mM KCl, 1 mM MgCl2, 50 μg/ml κ-casein, pH 7.7) and mixed thoroughly. Purified protein (either GFP-TPX2 or BuGZ-BFP) was precleared of aggregates by centrifugation at 80000 RPM for 10 min at 4°C in a TLA-100 rotor. The supernatant was added up to a final target protein concentration and a final seed dilution of 1/100 or 1/1000. The mixture was mixed twice with a p20 pipette and pipetted into a flow chamber. The flow chamber was sealed with nail polish and incubated coverslip side down for 10 minutes to allow bundles to settle. Bundles were imaged using 2-color TIRF. Exposure times and laser power were consistent across all tested concentrations for each protein. While the bundles are imaged on the glass coverslip, they form in the bulk region of the flow chamber before they settle to the glass surface.

For live snapping assays, coverslip-bottomed culture well plates were washed three times with assay buffer. lMicrotubule seeds were added, mixed thoroughly, and then imaged using oblique TIRF, well above the plane of the coverslip to avoid surface interactions. Once a good field of view was established, acquisition was started on the 647 channel only to maximize frame rates. Precleared GFP-TPX2 was pipetted into the well up to a final 500 nM concentration and a final seed dilution of 1/100 and mixed once with a p20 pipette. Acquisition was stopped after 5-10 min. Microtubule lengths were estimated using the straight line tool in Fiji (ImageJ) on the frame when the microtubules were best in focus.

All assays were done on a Nikon Ti-E microscope with a 100x objective and 1.49 numerical aperture. An ORCA-Fusion BT digital CMOS camera was used for acquisition.

The average structure factor $S(k)$ was calculated from the microtubule intensity channels for each MAP concentration. For each image $I(x, y)$, the power spectrum $P(k_x, k_y) = |\mathcal{F}[I(x, y)]|^2$ is computed, where $\mathcal{F}$ is the two-dimensional Fourier transform. $P(k_x, k_y)$ is converted to polar coordinates $P(k, \theta)$ and then $S(k)$ is calculated via $S(k) = \frac{1}{2\pi}\int_0^{2\pi} \mathrm{d}\theta\ P(k, \theta)$. The order parameter is then defined as $\frac{\int_\Omega S(k)dk}{\int_{-\infty}^{\infty} S(k)dk}$, which represents the relative power of the modes Ω that characterize the bundling transition (Fig. S2).

bioRxiv preprint doi: https://doi.org/10.64898/2026.06.19.733462; this version posted June 20, 2026. The copyright holder for this preprint (which was not certified by peer review) is the author/funder, who has granted bioRxiv a license to display the preprint in perpetuity. It is made available under a CC-BY 4.0 International license.

S2.5 *In vitro* atomic force microscopy assay on microtubules

Short, GMPCPP-stabilized microtubule seeds were diluted 1/10 in BRB80 and electrostatically adhered to an atomically smooth mica surface pre-incubated with a 1 M $MgCl_2$ solution for 10 min [18]. Unbound seeds were washed off with fresh BRB80, and the final solution with the desired protein concentration was pipetted onto the mica surface. Samples were probed with a silicon-nitride AFM cantilever tip (MLCT-BIO DC Bruker) with a nominal radius of 20 nm. Since the silicon nitride tip is negatively charged at the pH of BRB80 [19], analogous to the microtubule lattice, we assume that condensates which wet microtubules would similarly wet the AFM tip. Using the JPK NanoWizard 4 atomic force microscope, we first localized microtubules by generating elevation maps using tapping mode (Peakforce QNM). Then, using force spectroscopy mode, we performed a series of slow force ramps at specific locations along the length of a chosen microtubule in order to measure interfacial forces directly on the microtubule lattice. The force measurement ramps were carried out at velocity of 0.5 μm/s over 0.1 μm, with a force threshold of 0.2 nN.

The force curves were processed as follows. All force curves were shifted by a constant value equal to the average force between 98 and 100 nm from the surface so that the baseline was at a force of 0 pN. Some force curves (not plotted) were rejected if their baseline was not flat, judged by applying a cutoff of 0.02 nN difference between 90 nm and 100 nm. If the difference in the baseline far from the surface exceeded this value, it was assumed that the beginning of the ramp was not sufficiently quiescent for reliable force measurements. No filters or other smoothing of the raw data was applied. The value of the adhesion force for each force ramp was calculated by taking the minimum of the force curve. To calculate the effective surface tension, the minima of the average curves was used to extract an average adhesion force at contact. The adhesion energy was calculated by integration of the force over distance. In order to minimize errors from integrating over the baseline regions where noise might bias the integral, a linear subtraction fitted to the data between 60 and 100 nm was performed from the cumulative integral. The adhesion energy was calculated as the minimum of this corrected cumulative integral.

S2.6 *In vitro* atomic force microscopy assay on bulk droplets

To supplement the microtubule-specific measurements that quantify capillary forces directly on the microtubule lattice, measurements are made directly on large ($\sim \mu$m scale) condensate droplets sedimented onto a glass substrate to estimate condensate/water surface tension, γ, and the condensate viscosity, μ.

The experiment begins at $t = 0$ when condensate are diluted from a high salt storage buffer with BRB80 buffer to arrive at a final concentration of 4 μM in a glass bottomed petri dish (uncoated, ibidi μ-Dish 35 mm, low). Using a Zeiss LSM 900 / Axio Observer 7 with a long focal distance air objective (Objective LD PN 63x/0.75 Korr Ph2), the

bioRxiv preprint doi: https://doi.org/10.64898/2026.06.19.733462; this version posted June 20, 2026. The copyright holder for this preprint (which was not certified by peer review) is the author/funder, who has granted bioRxiv a license to display the preprint in perpetuity. It is made available under a CC-BY 4.0 International license.

DirectOverlay functionality is used to co-localize the AFM tip (Bruker SAA-SPH-1UM, with spring constant $k \approx 0.25$ N/m and radius $R = 1\mu$m) and the droplets. Ten force ramp measurements are made on an individual droplet, and the process is repeated for 18 and 15 droplets for TPX2 and BuGZ, respectively. The force ramp is initiated on the first droplet once a droplet of sufficient size has sedimented within the microscope field of view and the cantilever position can be calibrated with DirectOverlay, about 18 minutes for TPX2 and 9 minutes for BuGZ. Between each droplet measurement, the DirectOverlay calibration is repeated to ensure proper alignment with the sedimented droplet of choice. Droplets are chosen at random within the field of view. Example images of droplets at the end of the experiment are shown for TPX2 and BuGZ in Fig. S6 (a,e). We note that due to the small droplet sizes, the contact angle of the droplets on the glass substrates were not able to be detected reliably.

The force ramp is conducted at a fixed piezo velocity of 10 μm/s with a ramp height of 4 μm up to a force threshhold of 4 nN. From the approach curve, one can fit the effective viscosity from the repulsive force upon approach to the rigid glass interface, properly accounting for the cantilever deflection. Here, the corrected distance between the cantilever tip and the solid surface, H, is computed by correcting for the cantilever deflection as estimated by the contact slope of the measured force, $F(x)$. We define x as the piezo displacement coordinate, with $x = 0$ defined as the position where the force reaches its maximum, F_0. Following [20], if m is the absolute value of the contact slope (considered to be in the compliant zone and representative of the effective spring constant of the cantilever tip in contact with the surface), the contact position x_c is estimated as $x_c = F_0/m$ and the cantilever deflection at each position is given as $F(x)/m$. Therefore, the value of H can be represented as

$$H = x - \frac{F(x)}{m} - x_c + \frac{F(x_c)}{m} \tag{S66}$$

from this H function, the velocity dH/dt can be computed directly from the time series data.

To extract viscosity, we use the lubrication forces of a sphere near a plane [20],

$$F = \frac{6\pi\mu R^2 \frac{dH}{dt}}{H} = \frac{\beta \frac{dH}{dt}}{H}, \tag{S67}$$

valid when $H \ll R$. We restrict the fit to the region in which the distance of the sphere to the glass plane is less than 500 nm ($0 < H < 500$ nm) or the closest point where the force is greater than 10 pN ($F > 10$ pN), so that the lubrication approximation is a decent approximation and the force magnitude is above noise.

$$\beta_{\text{fit}} = \text{mean}\left(\frac{FH}{\frac{dH}{dt}}\right) \tag{S68}$$

bioRxiv preprint doi: https://doi.org/10.64898/2026.06.19.733462; this version posted June 20, 2026. The copyright holder for this preprint (which was not certified by peer review) is the author/funder, who has granted bioRxiv a license to display the preprint in perpetuity. It is made available under a CC-BY 4.0 International license.

where the fit is taken only over the allowable range of H and F specified above.

The baselines are subtracted by the force value at $x = 200$ nm for buffer and $x = 2.0$ μm for the condensates TPX2 and BuGZ. Force curves are filtered out by assessing the drift in the baseline at $x = 2.45 - 2.5$ μm if the average force in this region is greater than 0.5 nN after baseline subtraction.

This procedure is repeated for each individual approach force curve to create a histogram in Fig. S6 (f-h), rejecting only fits that are greater than three standard deviations away from the mean. In the histogram, the segments of the bars are colored by the time at which each force ramp was initiated, with $t = 0$ corresponding to when the condensate is first formed via dilution (time errors for $t = 0$ could be at most 60 seconds from the estimate, but all other time shifts are extracted directly from metadata). The fitting procedure is also done on the averaged curves in Fig. S6 (b-d). First, the force curves averaged at each x, then the fitting procedure, including calculating H, is done for that averaged force curve.

For the control conditions of buffer without condensate, we see that in Fig. S6 (c), the viscosity of a water is effectively recovered. All measurements are made around a temperature of $T = 24°$C, where the viscosity of water is 0.91 mPas. The measured viscosity is averaged out to be 0.96 mPas (legend). While the average force curve closely reproduces the value of water, the histogram of individually fitted force curves illustrates the spread across force ramps. Due to the noise in the force curves, there is a distribution of values of the viscosity for different measurements, although the trends in the fitted viscosity do not appear to be a function of time, as would be expected for a simple fluid containing only water and salt. The actual viscosity of water is within one standard of deviation (1.1 mPas) of the average value extracted from all force curves (2.0 mPas).

For BuGZ and TPX2, on the other hand, we get a much larger fitted viscosity than water, within the range of viscosities reported for condensates but on the lower end. The viscosity fitted to the average force curves are 487 mPas and 480 mPas for TPX2 and BuGZ, respectively. For TPX2, the shape of the average force curve does not closely model that of the lubrication approximation, likely indicating that there are elastic contributions to the response due to the condensates viscoelasticity. On the other hand, BuGZ is much better behaved, and appears to have a force curve more consistent with the lubrication analysis assuming a Newtonian liquid. The histogram averages are for 303 mPas and 383 mPas, with standard deviation of 257 mPas and 546 mPas, for TPX2 and BuGZ, respectively. There is a clear trend in both condensate systems that at later times, larger viscosity values are measured, corresponding to droplet aging.

Next, we describe the procedure used for estimating the surface tension of the condensed droplets, γ, that have been sedimented onto glass. At contact, for a small, perfectly wetting droplet between a sphere in contact with a flat interface, the adhesive force is:

$$F = -4\pi\gamma R. \tag{S69}$$

We estimate the adhesive forces from the retraction force curve minimums for TPX2 and

bioRxiv preprint doi: https://doi.org/10.64898/2026.06.19.733462; this version posted June 20, 2026. The copyright holder for this preprint (which was not certified by peer review) is the author/funder, who has granted bioRxiv a license to display the preprint in perpetuity. It is made available under a CC-BY 4.0 International license.

BuGZ. Note there is an adhesive minimum also for the case of a buffer without condensate present, but this minimum occurs in close contact with the surface. On the other hand, the apparent distance of the capillary adhesion driven by condensate appears at larger effective distances from the interface ($H > 100$ nm). While the fact that the observed capillary adhesion minimums do not occur precisely at contact may call into question the validity of the above contact expression, the force minimum on retraction can nevertheless be used to give a rough estimate of the surface tension within the context of a contact capillary bridge between glass and the cantilever tip. For TPX2 and BuGZ, respectively, the force minimums are -0.65 nN and -0.37 nN, respectively. These correspond to a surface tension of $\gamma_{\text{bulk}} \approx 50$ μN/m and $\gamma_{\text{bulk}} \approx 30$ μN/m, respectively. While the BuGZ value is in perfect agreement, we measure about a factor of 4 lower interfacial tension for bulk TPX2 droplets compared to condensed TPX2 on microtubules. Therefore, in the case of condensed TPX2, subtle nanoscale effects are at play in setting the value of the interfacial tension on microtubules, such as interactions with C-terminal tubulin tails. Other discrepancies in the value for TPX2 may indicate non-idealities in the droplet geometry compared to the simplistic model, to non-equilibrium effects in the measurements, or to the unknown effective contact angles of the condensate wetting interfaces. Because of the droplets' small size, it is difficult to determine the contact angle they make with the glass surface.

S2.7 *In vitro* motor sliding assay

Silanized and biotinylated coverslips were prepared for this assay. Coverslips were sonicated in 3 M NaOH for 30 minutes, washed with MilliQ water, then sonicated in Piranha solution for 45 minutes. Coverslips were then washed with MilliQ water and spin dried. 2-3 drops of GOPTS (3-glycidyloxypropyl trimethoxysilane) were sandwiched between two coverslips and placed in a 75°C oven for 30 min. Coverslips were then separated, washed with acetone, then dried with nitrogen gas. 1 g of HO-PEG-NH2 and 100 mg of biotin-CONH-PEG-NH2 were mixed, sandwiched between two coverslips, and incuvated overnight in a 75°C oven. The next day coverslips were separated, sonicated in MilliQ water for 30 min, washed with MilliQ water, then spin dried. Coverslips were stored at 4°C and used within 1-2 months.

A flow channel was made using a silanzed and biotinylated coverslip and incubated with 5% Pluronic F-127 solution for 5 min. The channel was washed with BRB80 (80 mM PIPES, 1 mM MgCl2, 1 mM EGTA, pH 6.8) and then incubated with 0.1 mg/ml NeutrAvidin for 10 min. The channel was washed with BRB80 then incubated with long Alexa-568 biotin microtubule seeds diluted 1/500 in BRB80 for 10 minutes. The channel was washed with assay buffer (25 mM HEPES, 25 mM KCl, 1 mM MgCl2, pH 7.5; 1% v/v glycerol, 50μg/ml κ-casein, 10 mM BME, 1 μM Mg-ATP, 20 mM glucose) then incubated with 100 nM of Eg5-GFP for 1 min. The channel was washed with assay buffer then incubated with short Atto-647 microtubule seeds diluted 1/200 in assay buffer

bioRxiv preprint doi: https://doi.org/10.64898/2026.06.19.733462; this version posted June 20, 2026. The copyright holder for this preprint (which was not certified by peer review) is the author/funder, who has granted bioRxiv a license to display the preprint in perpetuity. It is made available under a CC-BY 4.0 International license.

for 3 min. The channel was washed with assay buffer, the final reaction mixture (assay buffer containing target BuGZ-BFP concentration, 100 nM Eg5-GFP, 5 mM Mg-ATP, 320 μg/ml glucose oxidase, 55 μg/ml catalase) was pipetted in, then the channel was sealed with nail polish. The reaction was imaged using 4-color TIRF on a Nikon Ti-E microscope with a 100x objective and 1.49 numerical aperture. An ORCA-Fusion BT digital CMOS camera or an Andor Zyla scientific CMOS camera was used for acquisition targeting around 2 seconds per frame.

References

[1] HM Princen. Capillary phenomena in assemblies of parallel cylinders: Iii. liquid columns between horizontal parallel cylinders. *Journal of Colloid and Interface Science*, 34(2):171–184, 1970.

[2] Yu-Guo Tao, Wouter K den Otter, JT Padding, JKG Dhont, and WJ Briels. Brownian dynamics simulations of the self-and collective rotational diffusion coefficients of rigid long thin rods. *The Journal of chemical physics*, 122(24), 2005.

[3] Pierre-Gilles Gennes, Françoise Brochard-Wyart, David Quéré, et al. *Capillarity and Wetting Phenomena: Drops, Bubbles, Pearls, Waves*. Springer, 2004.

[4] Amol Bedarkar and Xiang-Fa Wu. Capillary torque in a liquid bridge between two angled filaments. *Journal of applied physics*, 106(11), 2009.

[5] Alban Sauret, François Boulogne, Beatrice Soh, Emilie Dressaire, and Howard A Stone. Wetting morphologies on randomly oriented fibers. *The European Physical Journal E*, 38:1–9, 2015.

[6] Jacob N Israelachvili. *Intermolecular and Surface Forces*. Academic press, 2011.

[7] Paul J Flory. Thermodynamics of high polymer solutions. *The Journal of Chemical Physics*, 10(1):51–61, 1942.

[8] Maurice L Huggins. Solutions of long chain compounds. *The Journal of Chemical Physics*, 9(5):440–440, 1941.

[9] John W Cahn and John E Hilliard. Free energy of a nonuniform system. i. interfacial free energy. *The Journal of Chemical Physics*, 28(2):258–267, 1958.

[10] Daoyuan Qian, Thomas CT Michaels, and Tuomas PJ Knowles. Analytical solution to the flory–huggins model. *The Journal of Physical Chemistry Letters*, 13(33):7853–7860, 2022.

bioRxiv preprint doi: https://doi.org/10.64898/2026.06.19.733462; this version posted June 20, 2026. The copyright holder for this preprint (which was not certified by peer review) is the author/funder, who has granted bioRxiv a license to display the preprint in perpetuity. It is made available under a CC-BY 4.0 International license.

[11] Evan Spruijt, Joris Sprakel, Martien A Cohen Stuart, and Jasper van der Gucht. Interfacial tension between a complex coacervate phase and its coexisting aqueous phase. *Soft Matter*, 6(1):172–178, 2010.

[12] Jan TG Overbeek and Michael J Voorn. Phase separation in polyelectrolyte solutions. theory of complex coacervation. *Journal of Cellular and Comparative Physiology*, 49(S1):7–26, 1957.

[13] Pengfei Zhang, Nayef M Alsaifi, Jianzhong Wu, and Zhen-Gang Wang. Polyelectrolyte complex coacervation: Effects of concentration asymmetry. *The Journal of Chemical Physics*, 149(16), 2018.

[14] Charles E Sing and Sarah L Perry. Recent progress in the science of complex coacervation. *Soft Matter*, 16(12):2885–2914, 2020.

[15] Lukasz P Kozlowski. Ipc–isoelectric point calculator. *Biology direct*, 11:1–16, 2016.

[16] Peter Rice, Ian Longden, and Alan Bleasby. Emboss: the european molecular biology open software suite. *Trends in Genetics*, 16(6):276–277, 2000.

[17] Nobuyuki Otsu et al. A threshold selection method from gray-level histograms. *Automatica*, 11(285-296):23–27, 1975.

[18] Sagar U Setru, Bernardo Gouveia, Raymundo Alfaro-Aco, Joshua W Shaevitz, Howard A Stone, and Sabine Petry. A hydrodynamic instability drives protein droplet formation on microtubules to nucleate branches. *Nature physics*, 17(4):493–498, 2021.

[19] Kabin Lin, Zhongwu Li, Yi Tao, Kun Li, Haojie Yang, Jian Ma, Tie Li, Jingjie Sha, and Yunfei Chen. Surface charge density inside a silicon nitride nanopore. *Langmuir*, 37(35):10521–10528, 2021.

[20] Ahmad Darwiche, François Ingremeau, Yacine Amarouchene, Abdelhamid Maali, Isabelle Dufour, and Hamid Kellay. Rheology of polymer solutions using colloidal-probe atomic force microscopy. *Physical Review E—Statistical, Nonlinear, and Soft Matter Physics*, 87(6):062601, 2013.
